# Supplementary material for: Food transfers, electronic food vouchers and child nutritional status among Rohingya children living in Bangladesh
Source: PLoS One. 2020 Apr 29;15(4):e0230457. doi: 10.1371/journal.pone.0230457 (PMC7190090; doi:10.1371/journal.pone.0230457)
Supplement: S1 File — (DOCX) [file pone.0230457.s001.docx]

**October 16, 2018**

***CONFIDENTIAL: For Research Purpose only***

**FORCIBLY DISPLACED MYANMAR NATIONALS’ FAMILY AND LOCAL COMMUNITY HOUSEHOLD SURVEY**

(October-November 2018; Reference Expenditure/ Income Period: 1 October 2017-30 September 2018)

***Survey designed and supervised by: International Food Policy Research Institute (IFPRI)***

***and***

***Bangladesh Institute of Development Studies (BIDS)***

**Household/Family Questionnaire**

**Contents**

[Module A: Household Identification 3](#_Toc527456365)

[**Consent Form** 4](#_Toc527456366)

[**Definition of Household** 5](#_Toc527456367)

[Module B: Household Characteristics 8](#_Toc527456368)

[**Module B1: Demographics** 8](#_Toc527456369)

[**Module B2: Additional demographic questions** 11](#_Toc527456370)

[**Module B3: Illness, all household members** 13](#_Toc527456371)

[Module C: Housing and Sanitation 15](#_Toc527456372)

[**Module C1: Housing** 15](#_Toc527456373)

[**Module C2: Water and Sanitation** 17](#_Toc527456374)

[Module D: Household Food Security 19](#_Toc527456375)

[Module E: Coping Strategies 22](#_Toc527456376)

[Module F: Current Household Assets 25](#_Toc527456377)

[Module G: Income and Inputs 26](#_Toc527456378)

[**Module G1: Income from Agriculture** 26](#_Toc527456379)

[**Module G2: Non-Agricultural Enterprises** 31](#_Toc527456380)

[**Module G3: Economic Activities and Wage Employment** 33](#_Toc527456381)

[**Module G4: Other Income** 35](#_Toc527456382)

[Module H: Credit 37](#_Toc527456383)

[Module I: Consumption Expenditure 39](#_Toc527456384)

[**Module I1: Food consumption** 39](#_Toc527456385)

[**Module I2: Non-food Expenditure Annual Recall (October 1, 2017 – September 30, 2018)** 43](#_Toc527456386)

[Sub-module I2a: Non-food Expenditure Monthly Recall 43](#_Toc527456387)

[Sub-module I2b: Non-food Expenditure Annual Recall (October 1, 2017 – September 30, 2018) 44](#_Toc527456388)

[Module K: Histories 47](#_Toc527456389)

[**Module K1: Migration history (Rohingya only)** 47](#_Toc527456390)

[**Module K2: History prior to migration (Rohingya only)** 49](#_Toc527456391)

[**Module K3: Subjective wellbeing (ALL Households)** 49](#_Toc527456392)

[Module L: Protection (All households) 51](#_Toc527456393)

[Module M: Assistance 53](#_Toc527456394)

[**Module M1: Assistance, Bangladesh (host) households** 53](#_Toc527456395)

[**Module M2: Assistance, Rohingya households** 54](#_Toc527456396)

# Module A: Household Identification

|  | **Question** | **Response** |
| --- | --- | --- |
| **nationality** | Who are you interviewing?  *(Pre-coded before the start of the interview)* | **Code [nationality]** |
| **camp** | ROHINGYA: Camp (name and code)  *(Pre-coded before the start of the interview)* |  |
| **block** | ROHINGYA: Block (name and code)  *(Pre-coded before the start of the interview)* |  |
| **district** | BANGLADESHI: District (name and code)  *(Pre-coded before the start of the interview)* |  |
| **upazila** | BANGLADESHI: Thana/ Upazila (name and code):  *(Pre-coded before the start of the interview)* |  |
| **union** | BANGLADESHI: Union (name and code):  *(Pre-coded before the start of the interview)* |  |
| **village** | BANGLADESHI: Village (name and code):  *(Pre-coded before the start of the interview)* |  |
| **household_id** | Household Identification Number: |  |
| **enumeratorname** | Name of Interviewer and code:  *(Pre-coded before the start of the interview)* |  |
| **supervisor** | Name of Supervisor and code:  *(Pre-coded before the start of the interview)* |  |

## **Consent Form**

Good morning/afternoon. I am ________ from the **Bangladesh Institute of Development Studies (BIDS)**, a Bangladeshi research organization based in Dhaka. Together with the **International Food Policy Research Institute (IFPRI)** and a team from the **Standardized Monitoring and Assessment of Relief and Transitions (SMART)**, we are conducting a survey that will provide our partners with necessary information to ultimately promote the welfare of host communities as well as displaced population from Myanmar. This project, funded by the **World Food Programme (WFP),** is the follow-up of last year’s **Rohingya Emergency Vulnerability Assessment (REVA)**. Your household has been chosen by a random selection process.

We are inviting you to be a participant in this study. We value your opinion and participation. There are no wrong answers to the questions we will be asking in the interview. We will use approximately 2 hours of your time to collect all the information. We will conduct the interview in separate phases: along with this visit from the **BIDS** team, a team from the **SMART** group will take anthropometric measures.

There will be no cost to you other than your time. There will be no risk as a result of your participating in the study. Your participation in this research is completely voluntary and there will not be any direct benefit for you. Your participation will not affect, in any form, your status of receiving aid. You are free to withdraw your consent and discontinue the interview at any time. You also have the right to refuse to answer specific questions or to not participate in the weight and height measuring. Not participating will not affect you or your family in any way.

This study is conducted anonymously. You will only be identified through code numbers. Your identity will not be stored with other information we collect about you. Your responses will be assigned a code number, and the list connecting your name with this number will be kept safely and will be destroyed once all the data has been collected and analyzed. Any information we obtain from you during the research will be kept strictly confidential.

Your participation will be highly appreciated. The answers you give will help provide better information to policy-makers, practitioners and program managers so that they can plan for better services that will respond to your needs.

You can ask questions concerning the study at any time during the interview or later. If you have questions or concerns about the study later, contact Olivette Burton, IFPRI IRB Coordinator, at O.Burton@cgiar.org; or the IFPRI office in Washington DC +1 202 862 4645.

The researcher read to me orally the consent form and explained to me its meaning. I agree to take part in this research. I understand that I am free to discontinue participation at any time if I so choose, and that the investigator will gladly answer any question that arise during the course of the research.

Please tick mark on the right box depending on the respondent’s consent

Consent given:

Contact People:

Principal BIDS Investigator (PI): Dr. Binayak Sen Principal IFPRI Investigator (PI): Dr. Paul Dorosh

Address: E-17 Agargaon, Sher-e- Bangla Nagar, Dhaka 1207 Address: 1201 Eye St, NW Washington, DC 20005

No

Yes

Tel: 880-2-8141722; E-mail of PI: b.sen@cgiar.org Tel: 1-202-862-8104; E-mail of PI: p.dorosh@cgiar.org

**Signature of the Enumerator: _____________ Date: /_____/_____/_____/**

## **Definition of Household**

A household is a group of people who live together and take food from the “same pot.” In our survey, a household member is someone who has lived in the household at least 6 months, and at least half of the week in each week in those months. In case of refugee/ displaced population from Myanmar complex context of migration needs to be kept in view in the specific camp conditions. In the latter’s case, WFP Food Card may be consulted to identify the household members.

Even those persons who are not blood relations (such as servants, lodgers, or agricultural laborers) are members of the household if they have stayed in the household at least 3 months of the past 6 months and take food from the “same pot.” If someone stays in the same household but does not bear any costs for food or does not take food from the same pot, they are not considered household members. For example, if two brothers stay in the same house with their families but they do not share food costs and they cook separately, then they are considered two separate households.

Generally, if one person stays more than 3 months out of the last 6 months outside the household, they are not considered household members. We do not include them even if other household members consider them as household members.

Exceptions to these rules should be made for:

Consider as household member

- A newborn child less than 3 months old.
- Someone who has joined the household through marriage less than 3 months ago.
- Servants, lodgers, and agricultural laborers currently in the household and will be staying in the household for a longer period but arrived less than 3 months ago.

Do not consider as household member

- A person who died very recently though stayed more than 3 months in last 6 months.
- Someone who has left the household through marriage more than 3 months ago.
- Servants, lodgers, and agricultural laborers who stayed more than 3 months in last 6 months but left permanently.

This definition of the household is very important. The criteria could be different from other studies you may be familiar with, but you should keep in mind that you should not include those people who do not meet these criteria.

**Module A Codes:**

| **nationality:** | |
| --- | --- |
| 1 | Rohingya |
| 2 | Bangladeshi |

| **strata:** | |
| --- | --- |
| 1 | Registered camp |
| 2 | Makeshift camp |
| **camp:** | |
| **block:** | |

If Nationality = 1 If Nationality = 2

| **district:** |
| --- |
| **upazila:** |
| **union:** |
| **district:** |
| **village:** |

| **Household ID:** |
| --- |
|  |

| **Sl no** | **Household Identification** | **Response** |
| --- | --- | --- |
| **date_today** | Date of the First visit (dd/mm/yy): |  |
| **date_previous** | Date of the second visit (dd/mm/yy): |  |
| **name** | Name of the Respondent: |  |
| **gender_resp** | Gender of respondent |  |
| **wh_head_hh** | Relationship with HH head | **Code [hh_roster]** |
| **head_name** | What is the head’s name?  *(If respondent is not the household head)* |  |
| **phone** | Mobile phone number |  |

# Module B: Household Characteristics

## **Module B1: Demographics**

| **B1_1** | How many people live in this household, including yourself? | | | | | | |  | | | | | | | |
| --- | --- | --- | --- | --- | --- | --- | --- | --- | --- | --- | --- | --- | --- | --- | --- |
| Mem  ID | Name | Gender  **code [gender]** | Age**  *In years* | Age (in total) months  *(If under 5 years)* | Is this person considered a household member under our definition and/or SMART's definition?  **Code [hh_**  **definition]** | Relation  to  Household Head  **Code [hh_**  **roster]** | Marital status  **Code [marital_status]** | Current main occupation  *(If older than 5)*  **Code [occupation]** | How many days in the previous week did [name] attend a learning facility?  *(If the person is reported as being a student in code for main occupation)* | Education  (Highest grade passed)  **Code [education]** | What subject/  type of education did [name] receive?  **Code [edu2]** | What type of learning center has this person most recently attended or is currently attending?  **Code [type_**  **school]** | For Children aged 6-18 years (whose main occupation is *not* student) | | |
|  |  |  |  |  |  |  |  |  |  |  |  |  | Has this person ever attended a learning center?  **Code [noyes]**  *(if no, skip to next person)* | Which year did [name] stop attending a school/ learning center? | What is the main reason that [name] stopped attending a school/ learning center?  **Code [reason_ stopschool]** |
| **ID** | **B1_2_name_hh_mem** | **B1_3_gender_hh_mem** | **B1_4_age_hh_mem** | **B1_4_age_under5** | **hh_definition** | **B1_5** | **B1_6** | **B1_7** | **B1_7b** | **B1_8a** | **B1_8b** | **B1_9** | **B1_10** | **B1_11** | **B1_12** |
| 1 |  |  |  |  |  |  |  |  |  |  |  |  |  |  |  |
| 2 |  |  |  |  |  |  |  |  |  |  |  |  |  |  |  |
| 3 |  |  |  |  |  |  |  |  |  |  |  |  |  |  |  |
| 4 |  |  |  |  |  |  |  |  |  |  |  |  |  |  |  |
| 5 |  |  |  |  |  |  |  |  |  |  |  |  |  |  |  |
| 6 |  |  |  |  |  |  |  |  |  |  |  |  |  |  |  |
| 7 |  |  |  |  |  |  |  |  |  |  |  |  |  |  |  |
| 8 |  |  |  |  |  |  |  |  |  |  |  |  |  |  |  |
| 9 |  |  |  |  |  |  |  |  |  |  |  |  |  |  |  |
| 10 |  |  |  |  |  |  |  |  |  |  |  |  |  |  |  |

Note: ^**^Write complete years. For example if age is 18 years and 9 months, write only 18 years.

**Module B1 Codes:**

| **gender:** | |
| --- | --- |
| 0 | Male |
| 1 | Female |

| **occupation:** | | | |
| --- | --- | --- | --- |
| 1 | Student | 39 | Plumber |
| 2 | Housewife | 40 | Electrician |
| 3 | Jobless | 41 | Carpenter |
| 4 | Retired | 42 | Mason |
| 5 | Child(age <5 no study/ work) | 43 | Doctor |
| 6 | Agricultural day labor | 44 | Village doctor |
| 7 | Earth work (government program) | 45 | Midwife |
| 8 | Earth work (other) | 46 | Herbal doctor/Kabiraj |
| 9 | Sweeper | 47 | Engineer |
| 10 | Scavenger | 48 | Lawyer/deed writer/Moktar |
| 11 | Tea garden worker | 49 | Religious leader (Imam/Muazzem/ Khadem/Purohit) |
| 12 | Construction labor | 50 | Lodging master |
| 13 | Factory worker | 51 | Private tutor/house tutor |
| 14 | Transport worker (bus/truck helper) | 52 | Beggar |
| 15 | Apprentice | 53 | Food Processing |
| 16 | Other wage labor (specify) | 54 | Small industry |
| 17 | Government/ parastatal | 55 | Handicrafts |
| 18 | Private enterprise (sales person, manager) | 56 | Small trader (roadside stand or stall) |
| 19 | NGO worker | 57 | Medium trader (shop or small store) |
| 20 | House maid | 58 | Large trader (large shop or whole sale) |
| 21 | Teacher (GoB-Primary school) | 59 | Fish Trader |
| 22 | Teacher(Non GoB Primary school) | 60 | Contractor |
| 23 | Teacher (GoB High school) | 61 | Milk collector |
| 24 | Teacher (Non-GoB High school) | 62 | Vet medicine seller |
| 25 | Teacher (college, university) | 63 | Feed supplier |
| 26 | Other salaried worker(specify) | 64 | Commercially feed producer |
| 27 | Rickshaw/van pulling | 65 | Animal Breeder |
| 28 | Driver of motor vehicle | 66 | Veterinary/paravet doctor |
| 29 | Tailor/seamstress | 67 | Working own farm (crop) |
| 30 | Potter | 68 | Share cropper/tenant |
| 31 | Blacksmith | 69 | Homestead farming |
| 32 | Cobbler | 70 | Fisherman (non owned/not leased water body) |
| 33 | Hair cutter | 71 | Raising fish / fish pond |
| 34 | Clothes washer | 72 | Raising poultry |
| 35 | Porter | 73 | Raising livestock |
| 36 | Goldsmith/silversmith | 74 | Milk producer |
| 37 | Repairman (appliances) | 75 | Other self employment |
| 38 | Mechanic (vehicles) | 76 | Physically/mentally challenged |

| **education:** | |
| --- | --- |
| 0 | No formal education |
| 1 | Preschool class |
| 2 | Reads in class I |
| 3 | Completed class I |
| 4 | Completed class II |
| 5 | Completed class III |
| 6 | Completed class IV |
| 7 | Completed class V |
| 8 | Completed class VI |
| 9 | Completed class VII |
| 10 | Completed class VIII |
| 11 | Completed class XIX |
| 12 | Completed secondary school or equivalent |
| 13 | Completed high school or equivalent |
| 14 | BA/BSC pass or equivalent |
| 15 | BA/BSC or equivalent (4 years undergraduate / honors degree) |
| 16 | Masters/Medical and above or equivalent |
| 17 | Too young to attend school |
| 18 | Other (specify) |

| **hh_roster:** | |
| --- | --- |
| 1 | Household Head |
| 2 | Household head’s Husband/wife |
| 3 | Son/daughter |
| 4 | Daughter/son -in-law |
| 5 | Grandson/daughter |
| 6 | Father/mother |
| 7 | Brother/sister |
| 8 | Niece/Nephew |
| 9 | Household Head’s cousin |
| 10 | Father-in-law/mother-in-law |
| 11 | Brother/Sister-in-law |
| 12 | Husband/wife’s niece/nephew |
| 13 | Household Head’s husband/wife’s cousin |
| 14 | Other relative |
| 15 | Permanent servant |
| 16 | Other Non relative/friends |

| **marital_status:** | |
| --- | --- |
| 1 | Unmarried (never married) |
| 2 | Married |
| 3 | Widow/widower |
| 4 | Divorced |
| 5 | Separated/Deserted |

| **edu2:** | |
| --- | --- |
| 1 | Conventional education |
| 2 | Vocational or Technical |
| 3 | Teacher's college |
| 4 | Nursing or paramedics |
| 5 | Religious studies |
| 6 | Other (specify) |
| 9998 | Not applicable |

| **noyes:** | |
| --- | --- |
| 0 | No |
| 1 | Yes |

| **type_school:** | |
| --- | --- |
| 1 | NGO school (BRAC) |
| 2 | Learning center managed by NGOs/UNICEF |
| 3 | Religious school (Madrasa) - Informal |
| 4 | Religious school (Madrasa) - Formal |
| 5 | Religious Centers (maktob, mosque-based) |
| 6 | Conventional school - Public |
| 7 | Conventional school - Private |

| **reason_stopschool:** | |
| --- | --- |
| 1 | Below school/madrasa age |
| 2 | Sick/disabled child |
| 3 | Child didn’t want to attend school |
| 4 | Teachers do not teach well |
| 5 | No latrine facility |
| 6 | Parents don’t want to send children to school |
| 7 | No school/madrasa nearby |
| 8 | Transport/communication problem |
| 9 | No ability/ Cannot bear expenses / No ability to buy school uniform |
| 10 | Children must work elsewhere for income |
| 11 | Engaged in family business/agriculture |
| 12 | Children need to take care of household chores/relatives |
| 13 | Insufficient amount of stipend money /education allowance |
| 14 | Not getting stipend, so withdrawn from school/madrasa |
| 15 | Don’t like to send girls to school |
| 16 | There are no female teacher in school |
| 17 | No only boys’ or only girls’ school |
| 18 | Boys tease girls/don’t like girls |
| 19 | Environment of school is not safe |
| 20 | No separate latrine facility for female students |
| 21 | Due to marriage |
| 22 | Had to leave Myanmar |
| 23 | No opportunity to study in Bangladesh |
| 24 | No facility |
| 25 | Facility too crowded/not suitable |
| 26 | Lack of teachers |
| 27 | School too far away |
| 28 | Health conditions do not allow |
| 29 | Family does not see the need |
| 30 | Just arrived but planning to attend |
| 31 | Safety risks |
| 32 | Other (specify) |

| **hh_definition:** | |
| --- | --- |
| 1 | SMART Survey only |
| 2 | REVA Survey only |
| 3 | Both Surveys |

## **Module B2: Additional demographic questions**

|  | **Question** | **Note** | **Response** |
| --- | --- | --- | --- |
| **B2_2** | How many female members fall under the following categories?  **For enumerators: if none, please input 0** | a. Single with child(ren)  b. Disability (body and /or mind)  c. Chronically ill or serious medical condition (ill for >2months)  d. Unaccompanied minors (girls <15) | a.  b.  c.  d. |
| **B2_4** | How many male members fall under the following categories?  **For enumerators: if none, please input 0** | a. Single with child(ren)  b. Disability (body and/or mind)  c. Chronically ill or serious medical condition (ill for >2months)  d. Unaccompanied minors (boys <15) | a.  b.  c.  d. |
| **B2_5** | Are there children < 18 separated from their parents living with you (unaccompanied minors)? If so, how many? | a. Female:  b. Male: | a.  b. |
| **B2_6** | If any children<18 separated from their parents are living with you, what are the 1 or 2 main reason(s)? | **Code [reason_childrenseparate]** |  |
| **B2_7a** | Has your household ever hosted (other) displaced population? | **Code [noyes]**  *(if no (0), skip to Module B3)* |  |
| **B2_7b** | When did the displaced population who you hosted arrive in Bangladesh? (can select multiple) | **Code [host_displ]** |  |
| **B2_8** | Are these people still living in your household? | **Code [noyes]**  *(if no (0), skip to Module B3)* |  |
| **B2_9** | Are they planning to stay or move on in the near future? | **Code [play_stay]** |  |

**Module B2 Codes:**

| **reason_childrenseparate** | |
| --- | --- |
| 1 | Sent by parent(s) |
| 2 | They do not have any other caretakers (for example, parents deceased, separated from parent(s), etc.) |
| 3 | Prefer to stay with others |
| 4 | Economically beneficial |
| 5 | Other (specify) |

| **host_displ:** | |
| --- | --- |
| 1 | Arrived since August 2017 |
| 2 | Arrived before August 2017 |

| **noyes:** | |
| --- | --- |
| 0 | No |
| 1 | Yes |

| **plan_stay:** | |
| --- | --- |
| 1 | Stay in the near future |
| 2 | Move in the near future |

## **Module B3: Illness, all household members**

|  | In the last 30 days have you suffered from any illness?  **Code [noyes]**  *(if no (0), skip to next line/member)* | What illness/symptoms did you suffer from?  *Report top 3 major symptoms/disease*  **Code [illnesses]** | | | Did this person seek any medical treatment related to the health problem suffered in the last 30 days with one of these people?  **Code [medical_treatment]**  *(if no (0) skip to next line/member)* | What was the principal reason why they did not do so:  **Code [reason_nottreat]** |
| --- | --- | --- | --- | --- | --- | --- |
| **HH Mem ID** | **B3_2** | **B3_3** | | | **B3_4** | **B3_5** |
|  |  |  |  |  |  |  |
|  |  |  |  |  |  |  |
|  |  |  |  |  |  |  |
|  |  |  |  |  |  |  |
|  |  |  |  |  |  |  |
|  |  |  |  |  |  |  |
|  |  |  |  |  |  |  |
|  |  |  |  |  |  |  |

**Module B3 Codes:**

| **noyes:** | |
| --- | --- |
| 0 | No |
| 1 | Yes |

| **medical_treatment** | |
| --- | --- |
| 0 | Did not seek |
| 1 | Govt. Health Worker |
| 2 | NGO Health Worker |
| 3 | Homeopath |
| 4 | Ayurved/Kabiraji/Hekim |
| 5 | Other Traditional/ Spiritual/Faith Healer |
| 6 | Govt. Doctor (Govt. Facility) |
| 7 | Govt. Doctor (Private Facility) |
| 8 | Doctor from NGO Facility |
| 9 | Doctor from Private Facility |
| 10 | Salesman from Pharmacy |
| 11 | Family Treatment |
| 12 | Self Treatment |
| 13 | Other, specify |

| **illnesses:** | |
| --- | --- |
| 1 | Fever |
| 2 | Diarrhea |
| 3 | Cold |
| 4 | Dysentery |
| 5 | Gout/arthritis |
| 6 | Abdomen pain |
| 7 | Other type of pain |
| 8 | Injury |
| 9 | Blood pressure |
| 10 | Heart disease |
| 11 | Breathing trouble |
| 12 | Weakness |
| 13 | Dizziness |
| 14 | Pneumonia |
| 15 | Typhoid |
| 16 | Tuberculosis |
| 17 | Malaria |
| 18 | Cancer |
| 19 | Leprosy |
| 20 | Paralysis |
| 21 | Epilepsy |
| 22 | Scabies |
| 23 | Kidney Diseases |
| 24 | Gall stone Diseases |
| 25 | Jaundice |
| 26 | Gastric /Ulcer |
| 27 | Diabetic |
| 28 | Female diseases |
| 29 | Pregnancy related |
| 30 | Other (specify) |

| **reason_nottreat** | |
| --- | --- |
| 1 | Too far |
| 2 | Too expensive |
| 3 | No proper treatment/medicine |
| 4 | Don't know where to go |
| 5 | No time |
| 6 | Preferred other options |
| 7 | Condition not serious enough |
| 8 | Other (specify) |

# Module C: Housing and Sanitation

## **Module C1: Housing**

|  | **Question** | **Note** | **Response** |
| --- | --- | --- | --- |
| **C1_1** | Do you or your household own or rent this dwelling?  *(if rent (1), skip to C1_5, if own (2), skip to C1_3)* | **Code [housing_situ]** |  |
| **C1_2** | Were you asked for a fee to set up your shelter/dwelling  *(if no (0), skip to C1_3)* | **Code [noyes]** |  |
| **C1_2b** | How much did you have to pay? | Taka |  |
| **C1_3** | Who mainly helped construct the house? | **Code [help_constr_house]** |  |
| **C1_4** | Estimate the monthly rent you could receive if you rented this dwelling or one exactly like it to another person?  *(If respondent does not pay rent)* | Taka  9997 = don’t know |  |
| **C1_5** | How much monthly rent do you pay for this dwelling?  *(If respondent pays rent)* | Taka |  |
| **C1_5b** | How do you pay your rent?  *(If respondent pays rent)* | **Code [way_rent]** |  |
| **C1_6** | *OBSERVE:*  What type of dwelling does the household live in? | **Code [type_dwelling]** |  |
| **C1_7** | *OBSERVE:*  The outer walls of the main dwelling of the household are predominantly made of what material? | **Code [material_dwelling]** |  |
| **C1_8** | *OBSERVE:*  The roof of the main dwelling is predominantly made of what material? | **Code [material_dwelling]** |  |
| **C1_9** | *OBSERVE:* The floor of the main dwelling is predominantly made of what material? | **Code [material_dwelling]** |  |
| **C1_10** | How many rooms (including those divided by simple partitions and including kitchen) are there in your shelter/home? | Number (Exclude rooms used for business) |  |
| **C1_11** | How many rooms are used for sleeping? | Number |  |
| **C1_13** | What is your main source of energy for cooking? | **Code [energy_cooking]** |  |
| **C1_14** | How do you mainly access firewood? | **Code [access_firewood]** |  |
| **C1_15** | What is your main source of lighting fuel? | **Code [lighting_fuel]** |  |

| **material_dwelling:** | |
| --- | --- |
| 1 | Concrete/Brick |
| 2 | Tin/CI Sheet |
| 3 | Wood |
| 4 | Mud |
| 5 | Bamboo |
| 6 | Bamboo and plastic/polythene |
| 7 | Jute straw |
| 8 | Plastic /Polythene |
| 9 | Cardboard/paper |
| 10 | Golpaata/Palm leaf |
| 11 | Grass/Straw |
| 12 | Other (specify) |

**Module C1 Codes:**

| **housing_situ:** | |
| --- | --- |
| 1 | Rent |
| 2 | Own |
| 3 | Do not own and live for free |
| 4 | Other (specify) |

| **noyes:** | |
| --- | --- |
| 0 | No |
| 1 | Yes |

| **help_constr_house:** | |
| --- | --- |
| 1 | Only myself |
| 2 | Myself and other family members |
| 3 | Friends |
| 3 | Other refugees living in the same camp helped |
| 4 | Labor from outside the camp helped |
| 5 | Government contractor helped |
| 6 | Others helped |

| **way_rent:** | |
| --- | --- |
| 1 | In-kind |
| 2 | Cash |
| 3 | Both |

| **type_dwelling:** | |
| --- | --- |
| 1 | No sign of damage |
| 2 | Slightly damaged |
| 3 | Somewhat damaged |
| 4 | Very damaged |
| 5 | In a very poor state |

| **energy_cooking:** | |
| --- | --- |
| 1 | Electricity |
| 2 | Supply gas |
| 3 | LPG |
| 4 | Kerosene |
| 5 | Firewood |
| 6 | Dried cow dung |
| 7 | Coal |
| 8 | Rice bran/saw dust/compressed rice husk |
| 9 | Dried leaves |
| 10 | Other (specify) |

| **access_firewood:** | |
| --- | --- |
| 1 | Buying |
| 2 | Gathering/collecting |
| 3 | Friends/relatives |
| 4 | Borrow |
| 5 | Relief/charity |
| 6 | Other (specify) |

| **lighting_fuel:** | |
| --- | --- |
| 1 | Electricity |
| 2 | Private Generator |
| 3 | Solar electricity |
| 4 | Kerosene |
| 5 | Candles |
| 6 | Torch/fire skewer |
| 7 | Others (specify) |
| 9998 | Not applicable/don't have any lighting |

## **Module C2: Water and Sanitation**

Note: Data collected from Household head

|  | **Question** | **Note** | **Response** |
| --- | --- | --- | --- |
| **C2_1** | Where do you mainly obtain your drinking water? | **Code [water_source]** |  |
| **C2_2** | What is the main source of water used for other purposes than drinking? | **Code [water_source]** |  |
| **C2_3** | Do you face any problems accessing water?  *(if no (0), skip to C2_5)* | **Code [noyes]** |  |
| **C2_4** | What are the two main problems?  *Allow for up to 2 responses* | **Code [problems_water]** |  |
| **C2_5** | Main drinking water purifying process | **Code [purifying_water]** |  |
| **C2_6** | What is the main type of latrine that you use? | **Code [latrine_type]** |  |
| **C2_7** | How many other households (aside from yours) use the same latrine? |  |  |
| **C2_9** | What is the main way that household members age 5 years and above wash hands after defecation? | **Code [washhands]** |  |
| **C2_10** | Do you face any sanitation problems?  *(if no(0), skip to Module D)* | **Code [noyes]** |  |
| **C2_11** | What are the two main problems?  *Allow for up to 2 responses* | **Code [problems_sanitation]** |  |

**Module C2 Codes:**

| **water_source:** | |
| --- | --- |
| 1 | Piped water tap |
| 2 | Storage tanks tap |
| 3 | Tubewells/hand pump |
| 4 | Protected spring |
| 5 | Unprotected well |
| 6 | Unprotected spring |
| 7 | Surface water/puddle/pond/stream |
| 8 | Water truck |
| 9 | Bottled water/sachet |

| **latrine_type:** | |
| --- | --- |
| 1 | None (open field) |
| 2 | Kutcha (fixed place) |
| 3 | Pucca (unsealed) |
| 4 | Sanitary without flash (water sealed) |
| 5 | Sanitary with flash (water sealed) |
| 6 | Other (specify) |

| **noyes:** | |
| --- | --- |
| 0 | No |
| 1 | Yes |

| **problems_water:** | |
| --- | --- |
| 1 | Lack of sufficient water points |
| 2 | Water points not functioning |
| 3 | Waiting/Queuing time at water points |
| 4 | Distance to water points |
| 5 | Restricted access to water points |
| 6 | Lack of storage containers |
| 7 | Don’t like taste, quality etc |
| 8 | Safety/harassment reaching or at water point |

| **purifying_water:** | |
| --- | --- |
| 1 | Filtered |
| 2 | Boiled |
| 3 | Boiled and filtered |
| 4 | Chemically treated (iodine/chlorine) |
| 5 | UV treated |
| 6 | None |
| 7 | Others (specify) |

| **washhands:** | |
| --- | --- |
| 1 | Use only water |
| 2 | Use ash and water |
| 3 | Use mud and water |
| 4 | Wash only one hand with soap |
| 5 | Wash both hands with soap |

| **problems_sanitation:** | |
| --- | --- |
| 1 | Lack of sufficient facilities |
| 2 | Facilities not functioning |
| 3 | Waiting time at facilities |
| 4 | Distance to facilities |
| 5 | Restricted access to facilities |
| 6 | Safety/harassment reaching or at facilities |
| 7 | Other specify |
| 8 | None |

# Module D: Household Food Security

|  | **Question** | **Note** | **Response** |
| --- | --- | --- | --- |
| **D1_1** | How many meals did the adults (18+) in this household eat yesterday? | a. Female  b. Male | a.  b. |
| **D1_2** | How many meals did children between the ages of 6-17 eat yesterday? | a. Female  b. Male | a.  b. |
| **D1_3** | How many meals did children between the ages of 2-5 eat yesterday? | a. Female  b. Male | a.  b. |

|  | | **Question** | | **Note** | | **Response** |
| --- | --- | --- | --- | --- | --- | --- |
| **D1_4** | | In the next section, there are some questions that I want to ask to a female member of your household between the ages of 15-49. Which of these members will answer these questions? | | Will select one member from household roster  9998 = No woman in this age range available | |  |
| **Group**  **Number** | **Food items/groups** | | **Examples** | How many days **over the last 7 days**, did members of your household eat the following food items, prepared and/or consumed at home?  *If 0, skip to next item/line* | **(To enumerators: please identify one female (age 15-49) in the households and ask below questions)**  Did you eat the following food yesterday during the day or night?  **Code [noyes]** | **How was this food acquired?**  Write the main source of food for the past 7 days  **Code [acquire_food]** |
|  |  | |  | **D1_5** | **D1_6** | **D1_7** |
| 1 | **Cereals or tubers** | | **Rice, potato, naan etc.** |  |  |  |
| 2 | Foods made from grain | | Porridge, bread, rice, chapatti, roti, pasta/noodles or other foods made from grains |  |  |  |
| 3 | White roots and tubers and plantains | | Potato, white flesh sweet potato, taro and/or other tubers & root |  |  |  |
| 4 | **Pulses and groundnuts** | | **Moshul, khassir, etc.** |  |  |  |
| 5 | Pulses (beans, peas and lentils) | | Moshul, boot, motor, fresh or dried seeds, lentils, or bean/pea products including hummus, tofu and tempeh |  |  |  |
| 6 | Nuts and seeds | | Khassir, felon, any tree nut, groundnut / peanut, certain seeds, or nut/seed pastes |  |  |  |
| 7 | **Milk and milk products** | | **Fresh milk, powdered milk, yogurt, cheese, other dairy products** (exclude margarine/butter or small amounts of milk for tea / coffee) |  |  |  |
| 8 | **Eggs, meat, fish, shells** | | **Organ meat, flesh meat, fish, dried fish, eggs, etc.** |  |  |  |
| 9 | Organ meat | | Liver, kidney, heart, other organs |  |  |  |
| 10 | Meat and poultry | | Flesh meat: beef, lamb, goat, chicken, duck |  |  |  |
| 11 | Fish and seafood | | Fresh fish, shellfish, dried fish |  |  |  |
| 12 | Eggs | | Chicken eggs, duck eggs |  |  |  |
| 13 | **Vegetables** | | **Kolmi shak, puishak, carrots, spinach etc.** |  |  |  |
| 14 | Vitamin A-rich vegetables, roots  and tubers | | Carrot, red pepper, pumpkin |  |  |  |
| 15 | Dark green leafy vegetables | | Spinach, broccoli, amaranth, jute leaves, kolmi shak, puishak and/or other dark green leaves |  |  |  |
| 16 | Other vegetables | | Any other vegetables |  |  |  |
| 17 | **Fruits** | | **Mango, banana, etc.** |  |  |  |
| 18 | Vitamin A-rich fruits | | Mango, papaya, apricot, peach |  |  |  |
| 19 | Other fruits | | Banana, oranges, apples and any other fruits |  |  |  |
| 20 | **Sugar** | | **Sugar, honey, jam, cakes, candy, cookies, pastries, cakes and other sweet** (sugary drinks) |  |  |  |
| 21 | **Oil** | | **Vegetable oil, palm oil, shea butter, ghee, margarine, other fats / oil** |  |  |  |
| 22 | **Condiments** | | **Condiments / Spices, tea, coffee / cocoa, salt, garlic, spices, yeast / baking powder, tomato / sauce, meat or dry fish as a condiment, condiments including small amount of milk / tea coffee** |  |  |  |

**Module D Codes:**

| **noyes:** | |
| --- | --- |
| 0 | No |
| 1 | Yes |

| **acquire_food:** | |
| --- | --- |
| 1 | Purchase (cash) |
| 2 | Purchase (card) |
| 3 | Food assistance (Food distribution/GFD/in-kind) |
| 4 | Food assistance (food card/e-voucher) |
| 5 | Army distributing food |
| 6 | Support from relatives / friends |
| 7 | Barter and exchange |
| 8 | Borrowing |
| 9 | Begging / scavenging |
| 10 | Gathering of wild foods (plants / insects) |
| 11 | Hunting / fishing |
| 12 | Own production / vegetable garden |

# Module E: Coping Strategies

|  | **Question** | **Response** |
| --- | --- | --- |
| **E1_3** | In the last 7 days, how many days did your family have to… | Frequency (number of days from 0 to 7) |
| **1** | Rely on less preferred, less expensive food |  |
| **2** | Borrow food or relied on help from friends or relatives |  |
| **3** | Reduce the number of meals eaten per day |  |
| **4** | Reduce portion size of meals |  |
| **5** | Restrict consumption by adults for young children to eat |  |
| **6** | Send household members elsewhere to eat |  |
| **7** | Restrict consumption by women and prioritize other members of the household |  |
| **8** | Restrict consumption by men and prioritize other members of the household |  |
| **9** | Everyone in the household went a whole day without eating |  |

|  | **Question** |  |  |
| --- | --- | --- | --- |
| **E1_5** |  | During the past 30 days, did anyone in your household have to engage in any of the following behaviours due to a lack of food or a lack of money to buy food or meet other basic needs?  **Code [noyes]**  *(if yes (1), skip to next item/line)* | Why did you not engage this strategy in the last 30 days?  **Code [response_copingstrategies2]** |
|  |  | **E1_5** | **E1_5b** |
| **1** | Selling household goods (radio, furniture, mobile, solar panel, television, clothes, kitchen items, etc.) |  |  |
| **2** | Selling jewelry/gold |  |  |
| **3** | Spent savings |  |  |
| **4** | Bought food on credit |  |  |
| **5** | Borrowed money to buy food |  |  |
| **6** | Selling productive assets or means of transport (sewing machines, wheelbarrow, bicycle, livestock etc.) |  |  |
| **7** | Reduce essential non-food expenditures such as education, health and clothes |  |  |
| **8** | Withdrew children from school |  |  |
| **9** | Children (under 15 years old) are working to contribute to household income (e.g. maid, casual labour) |  |  |
| **10** | Children (15-17) are working long hours (>43 hours) or work in hazardous conditions |  |  |
| **11** | Adults (18+) are working long-hours (>43 hours) or in hazardous conditions |  |  |
| **12** | Marriage of children under 18 |  |  |
| **13** | Inter-marriage |  |  |
| **14** | Begging |  |  |
| **15** | Accept high risks, illegal temporary job |  |  |
| **16** | Sold house or land |  |  |
| **17** | Reduced expenses on agricultural, livestock or fisheries inputs |  |  |
| **18** | Depending on food rations and/or support from neighbors and relatives as only food/income source |  |  |
| **19** | Collection of firewood for selling (not usual income) |  |  |
| **20** | Selling sharing and exchanging food rations |  |  |
| **21** | Selling non-food items that were provided as assistance |  |  |
| **22** | Selling labor in advance |  |  |

**Module E Codes:**

| **noyes:** | |
| --- | --- |
| 0 | No |
| 1 | Yes |

| **response_copingstrategies2:** | |
| --- | --- |
| 1 | Because it was not necessary |
| 2 | Because I already sold those assets or exhausted this activity within the last 12 months and cannot continue to do it |
| 3 | N/A |

# Module F: Current Household Assets

| Description of asset | Asset code | Did you own this item three years ago?  **Code [noyes]** | Does your household own the item today?  **Code [noyes]**  *(if no (0), skip to next line/item)* | Quantity owned today | If you had to sell all of [...] today, how much would you receive for it?  *Taka* |
| --- | --- | --- | --- | --- | --- |
| **Item** |  | **F1_1** | **F1_2** | **F1_4** | **F1_5** |
| Table / chair |  |  |  |  |  |
| Bed / Khat / Chowki |  |  |  |  |  |
| Blankets |  |  |  |  |  |
| Floor mats |  |  |  |  |  |
| Mosquito net |  |  |  |  |  |
| Armoire/Cabinet/ Alna |  |  |  |  |  |
| Shelves/elevated storage |  |  |  |  |  |
| Buckets / Plastic Pots |  |  |  |  |  |
| Metal cooking pots |  |  |  |  |  |
| Water storage containers |  |  |  |  |  |
| Trunk /Suitcase |  |  |  |  |  |
| Kerosene/LPG stove |  |  |  |  |  |
| Mud stove |  |  |  |  |  |
| Electric fan |  |  |  |  |  |
| Wall clock /watch |  |  |  |  |  |
| Mobile phone set |  |  |  |  |  |
| Radio |  |  |  |  |  |
| Torch/flashlight |  |  |  |  |  |
| Other electrical devices (dvd player, television etc) |  |  |  |  |  |
| Jewelry (gold/silver) |  |  |  |  |  |
| Bicycle |  |  |  |  |  |
| Van (tricycle van) |  |  |  |  |  |
| Boat |  |  |  |  |  |
| Fishing net |  |  |  |  |  |
| Saw |  |  |  |  |  |
| Hammer |  |  |  |  |  |
| Spade (Kodal) |  |  |  |  |  |
| Axe (Kural) |  |  |  |  |  |
| Shabol |  |  |  |  |  |
| Daa |  |  |  |  |  |
| Spade/shovel |  |  |  |  |  |
| Pick axe |  |  |  |  |  |
| Machete/large knife |  |  |  |  |  |
| Reaper/Sickle |  |  |  |  |  |
| Shallow tube well |  |  |  |  |  |
| Solar energy panel |  |  |  |  |  |
| Electricity Generator |  |  |  |  |  |

# Module G: Income and Inputs

## **Module G1: Income from Agriculture**

|  | **Question** | **Response (in Decimals)** |
| --- | --- | --- |
| **G1_1a** | Total own cultivable land (including leased out/rented out/mortgaged out) |  |
| **G1_1b** | Own homestead (land only) |  |
| **G1_1c** | Own non-cultivable land |  |
| **G1_1d** | Leased in (government) |  |
| **G1_1e** | Share cropped out |  |
| **G1_1f** | Mortgaged out |  |
| **G1_1g** | Rented/leased out |  |
| **G1_1h** | Share cropped in |  |
| **G1_1i** | Rented/leased in |  |
| **G1_1j** | Mortgaged in |  |

| **G1_7_pre** | Did you or anyone in your household cultivate any following crops in the last 12 months? | **Code [noyes]**  *(if no (0), skip to G1_14_pre)* |  |
| --- | --- | --- | --- |

|  |  | Did your household cultivate this crop in the last 12 months?  **Code [noyes]**  *(if no (0), skip to next line/crop)* | How much land did you cultivate under this crop in the last 12 months?  *Decimals* | How much in total of [crop] did you produce in the last 12 months?  *Should equal the sum of G1_10, G1_11, G1_12, and G1_13* | | How much did your household consumed in the last 12 months?  *KG* | How much did your household sell in the last 12 months  *KG* | How much did your household stock in the last 12 months?  *KG* | How much did your household use in the last 12 months (given to the landlord, given to wages, used as seed, feed for animals?  *KG*  *If 0, skip to next line/crop* | How much of it was used for the following purposes in the last 12 months?  *Should sum to G1_13* | | | | | |
| --- | --- | --- | --- | --- | --- | --- | --- | --- | --- | --- | --- | --- | --- | --- | --- |
|  |  |  |  | Kg | Unit price in taka  (taka/kg) |  |  |  |  | Given to landlord  *KG* | Given for Wages  *KG* | Used as Seed  *KG* | Feed for animals  *KG* | Waste  *KG* | Other  *KG* |
| **Crop** | **Code** | **G1_7** | **G1_8** | **G1_9a** | **G1_9b** | **G1_10** | **G1_11** | **G1_12** | **G1_13** | **G1_13a** | **G1_13b** | **G1_13c** | **G1_13d** | **G1_13e** | **G1_13f** |
| Aus | 01 |  |  |  |  |  |  |  |  |  |  |  |  |  |  |
| Aman | 02 |  |  |  |  |  |  |  |  |  |  |  |  |  |  |
| Boro | 03 |  |  |  |  |  |  |  |  |  |  |  |  |  |  |
| Wheat | 04 |  |  |  |  |  |  |  |  |  |  |  |  |  |  |
| Maize | 05 |  |  |  |  |  |  |  |  |  |  |  |  |  |  |
| Jute | 06 |  |  |  |  |  |  |  |  |  |  |  |  |  |  |
| Sugarcane | 07 |  |  |  |  |  |  |  |  |  |  |  |  |  |  |
| Pulses | 08 |  |  |  |  |  |  |  |  |  |  |  |  |  |  |
| Oil Seed | 09 |  |  |  |  |  |  |  |  |  |  |  |  |  |  |
| **By Product** |  |  |  |  |  |  |  |  |  |  |  |  |  |  |  |
| By product of paddy | 11 |  |  |  |  |  |  |  |  |  |  |  |  |  |  |
| By product of wheat | 12 |  |  |  |  |  |  |  |  |  |  |  |  |  |  |
| By product of jute | 13 |  |  |  |  |  |  |  |  |  |  |  |  |  |  |
| By product of sugarcane | 14 |  |  |  |  |  |  |  |  |  |  |  |  |  |  |
| Potato | 15 |  |  |  |  |  |  |  |  |  |  |  |  |  |  |
| Onion | 16 |  |  |  |  |  |  |  |  |  |  |  |  |  |  |
| Garlic | 17 |  |  |  |  |  |  |  |  |  |  |  |  |  |  |
| Ginger | 18 |  |  |  |  |  |  |  |  |  |  |  |  |  |  |
| Turmeric | 19 |  |  |  |  |  |  |  |  |  |  |  |  |  |  |
| Tomato | 21 |  |  |  |  |  |  |  |  |  |  |  |  |  |  |
| Brinjal | 22 |  |  |  |  |  |  |  |  |  |  |  |  |  |  |
| Green Banana/Green Papaya | 23 |  |  |  |  |  |  |  |  |  |  |  |  |  |  |
| Cauliflower/Cabbage | 24 |  |  |  |  |  |  |  |  |  |  |  |  |  |  |
| Pumpkin | 25 |  |  |  |  |  |  |  |  |  |  |  |  |  |  |
| Radish | 26 |  |  |  |  |  |  |  |  |  |  |  |  |  |  |
| Green Chili | 27 |  |  |  |  |  |  |  |  |  |  |  |  |  |  |
| Bean | 28 |  |  |  |  |  |  |  |  |  |  |  |  |  |  |
| Patal | 29 |  |  |  |  |  |  |  |  |  |  |  |  |  |  |
| Lady's Finger | 31 |  |  |  |  |  |  |  |  |  |  |  |  |  |  |
| Puisak | 32 |  |  |  |  |  |  |  |  |  |  |  |  |  |  |
| Others (specify) | 33 |  |  |  |  |  |  |  |  |  |  |  |  |  |  |
| **Fruits** |  |  |  |  |  |  |  |  |  |  |  |  |  |  |  |
| Mango | 41 |  |  |  |  |  |  |  |  |  |  |  |  |  |  |
| Jackfruit | 42 |  |  |  |  |  |  |  |  |  |  |  |  |  |  |
| Ripe Banana | 43 |  |  |  |  |  |  |  |  |  |  |  |  |  |  |
| Ripe Papaya | 44 |  |  |  |  |  |  |  |  |  |  |  |  |  |  |
| Pineapple | 45 |  |  |  |  |  |  |  |  |  |  |  |  |  |  |
| Leechee | 46 |  |  |  |  |  |  |  |  |  |  |  |  |  |  |
| Melon/Bangi | 47 |  |  |  |  |  |  |  |  |  |  |  |  |  |  |
| Guava | 48 |  |  |  |  |  |  |  |  |  |  |  |  |  |  |
| Others (specify) | 49 |  |  |  |  |  |  |  |  |  |  |  |  |  |  |

| **LIVESTOCK AND POULTRY** | | | | | | | | | | | | | |
| --- | --- | --- | --- | --- | --- | --- | --- | --- | --- | --- | --- | --- | --- |
| **G1_14_pre** | | | | Did you or anyone in your household engage in any livestock activity the last 12 months? | | | | **Code [noyes]**  *(if no (0), skip to G1_16)* | | | |  | |
|  | | How many ..[NAME OF THE ANIMAL].. own? What is their total value?  *(if none, skip to next item/line)* | | | How many were born/did your household purchase in the last 12 months? | | How many died in the last 12 months? | | | How many did your household consume in the 12 months? | | How many did your household sell in the 12 months? | |
| **Animal/animal product** | **Code** | **Number** | **Value in Taka** | | **Number** | **Value in Taka** | **Number** | | **Value in Taka** | **Number** | **Value in Taka** | **Number** | **Value in Taka** |
|  |  | **G1_15a** | **G1_15b** | | **G1_15c** | **G1_15d** | **G1_15e** | | **G1_15f** | **G1_15g** | **G1_15h** | **G1_15i** | **G1_15j** |
| Cattle | 1 |  |  | |  |  |  | |  |  |  |  |  |
| Goat and sheep | 2 |  |  | |  |  |  | |  |  |  |  |  |
| Buffalo | 3 |  |  | |  |  |  | |  |  |  |  |  |
| Chicken and duck | 4 |  |  | |  |  |  | |  |  |  |  |  |
| Other Birds | 5 |  |  | |  |  |  | |  |  |  |  |  |
| Meat (cattle, buffalo) | 6 |  |  | |  |  |  | |  |  |  |  |  |
| Poultry | 7 |  |  | |  |  |  | |  |  |  |  |  |
| Mutton, sheep | 8 |  |  | |  |  |  | |  |  |  |  |  |
| Milk | 9 |  |  | |  |  |  | |  |  |  |  |  |
| Eggs | 10 |  |  | |  |  |  | |  |  |  |  |  |
| Animal skins | 11 |  |  | |  |  |  | |  |  |  |  |  |
| Cow dung | 12 |  |  | |  |  |  | |  |  |  |  |  |
| Other | 13 |  |  | |  |  |  | |  |  |  |  |  |

| **FISH FARMING AND FISH CAPTURE** | | | | | | | | | | |
| --- | --- | --- | --- | --- | --- | --- | --- | --- | --- | --- |
| **G1_16** | | Did you or anyone in your household engage in any fishing or fish farming in the last 12 months? | | | **Code [noyes]** *(if no (0), skip to G1_21)* | | | |  | |
|  | | How much did you produce (catch) in the past 12 months?  *(if none, skip to next item/line)* | | How much did your household sell in the past 12 months? | | | How much did your household consume in the last 12 months? | | How much does your household have in stocks? | |
| **Fish type** | **Code** | **Kg** | **Value in Taka** | **Kg** | | **Value in Taka** | **Kg** | **Value in Taka** | **Kg** | **Value in Taka** |
|  |  | **G1_18a** | **G1_18b** | **G1_19a** | | **G1_19b** | **G1_20a** | **G1_20b** | **G1_20c** | **G1_20d** |
| Fish farm | 1 |  |  |  | |  |  |  |  |  |
| Fish Hatchery | 2 |  |  |  | |  |  |  |  |  |
| Marine fishing | 3 |  |  |  | |  |  |  |  |  |
| Canal/river fishing | 4 |  |  |  | |  |  |  |  |  |
| Swampland/marsh/fen fishing | 5 |  |  |  | |  |  |  |  |  |
| Pond/sink fishing | 6 |  |  |  | |  |  |  |  |  |
| Fish fry, crab frog | 7 |  |  |  | |  |  |  |  |  |
| Dry fish | 8 |  |  |  | |  |  |  |  |  |
| Other, specify | 9 |  |  |  | |  |  |  |  |  |

| **FARM FORESTRY** | | | | | | | | | |
| --- | --- | --- | --- | --- | --- | --- | --- | --- | --- |
| **G1_21** | | Did you or anyone in your household engage in any farm forestry in the last 12 months? | | | **Code [noyes]** *(if no (0), skip to G1_26)* | | |  | |
|  | | How many trees do you presently own? What is their value?  *(if none, skip to next item/line)* | | How much did your household sell in the last 12 months? | | | How much did your household consume in the 12 months? | | |
| **Tree type** | **Code** | **Number** | **Value in Taka** | **Number** | | **Value in Taka** | **Number** | | **Value in Taka** |
|  |  | **G1_23a** | **G1_23b** | **G1_24a** | | **G1_24b** | **G1_25a** | | **G1_25b** |
| Bamboo | 1 |  |  |  | |  |  | |  |
| Timber trees | 2 |  |  |  | |  |  | |  |
| Fruit trees | 3 |  |  |  | |  |  | |  |
| Firewood trees | 4 |  |  |  | |  |  | |  |
| Honey/honeycomb | 5 |  |  |  | |  |  | |  |
| Sapling/seedling sale | 6 |  |  |  | |  |  | |  |
| Flower sale | 7 |  |  |  | |  |  | |  |
| Flower /fruit seed sale | 8 |  |  |  | |  |  | |  |
| Other, specify | 9 |  |  |  | |  |  | |  |

| **AGRICULTURAL INPUTS: (***Only asked if household has income from any agriculture activity list above)* | | | |
| --- | --- | --- | --- |
| Expenditure item and code |  | Did your household spend any money on the (item) in last 12 months?  **Code [noyes]**  *(if no (0), skip to next line/item)* | How much did your household spend on the (item) in the last 12 months?  *Taka* |
| **Items of expenditure** | **Code** | **G1_26** | **G1_27** |
| Seed seedling (crop seedling) | 1 |  |  |
| Seed (forest seedling) | 2 |  |  |
| Fertilizer (chemical) | 3 |  |  |
| Fertilizer (composed/mixed) | 4 |  |  |
| Food of draft animal (animals for crop cultivation *only*) | 5 |  |  |
| Tractor/ tiller/ power tiller (rental) | 6 |  |  |
| Irrigation expenses | 7 |  |  |
| Insecticides | 8 |  |  |
| Rent (agricultural land) | 10 |  |  |
| Carrying cost of goods and transportation expenses (agricultural goods) | 11 |  |  |
| Salary/wages of laborer employed in agriculture | 12 |  |  |
| Insurance expenses (agriculture related) | 13 |  |  |
| Interest of the Agriculture loan | 14 |  |  |
| Electricity and fuel cost | 15 |  |  |
| Bees culture expenses | 16 |  |  |
| Fish production expenses | 17 |  |  |
| Livestock rearing expenses (Treatment cost etc.) | 18 |  |  |
| Poultry rearing expenses (Treatment cost etc.) | 19 |  |  |
| Other (specify) | 20 |  |  |

## **Module G2: Non-Agricultural Enterprises**

|  | **Question** | **Note** | **Response** |
| --- | --- | --- | --- |
| **G2_1** | Did you or anyone in your household operate (either solely or jointly) any business enterprise in the last 12 months? | **Code [noyes]**  *(if no (0), skip to Module G3)* |  |
| **G2_2** | How many enterprises did your household operate in the last 12 months? | *If more than 2, will be asked only about the 2 most important.* |  |

|  | What kind of enterprise did/ do your household solely or jointly own and operate in the past 12 months?  **Code [kind_ent]** | Which people in the household work in this enterprise / activity?  *Will select from household roster* | Since when has this enterprise activity been operating?  *Month and year* | Where do you operate the enterprise/  activity?  **Code [place_ent]** | How many months did the enterprise / activity operate in the past 12 months? | What is the household’s share of this enterprise / activity? | What share of profit is owned by the household? | Who are your most important customers?  **Code [customer]**  *Select top 2* | Is the enterprise activity registered by the government or local gov’t?  **Code [noyes]** | What was your main source of finance for setting up the business?  **Code [source_finance]**  *Select top 2* |
| --- | --- | --- | --- | --- | --- | --- | --- | --- | --- | --- |
|  | **G2_3** | **G2_4** | **G2_5** | **G2_6** | **G2_7** | **G2_8** | **G2_9** | **G2_10** | **G2_11** | **G2_12** |
| **1** |  |  |  |  |  |  |  |  |  |  |
| **2** |  |  |  |  |  |  |  |  |  |  |

|  | How many employees were engaged in the past 12 months? | What problems if any have you had in running your businesses in the last 12 months?  **Code [problems_busi]**  *Can select up to 3* | Gross revenues from this enterprise in the last 12 months  *Taka* | Expenditures on … for this enterprise in the past 12 months  *Taka* | | | | | | So your net revenue for this enterprise in the last 12 months is about [..]?  **Code [noyes]**  *Auto-calculates from gross revenue and expenditures* | What is your approximate net revenue (take home profit)?  *If they responded “no” to previous question* | Expenditures on capital goods  *Taka* | Sales of assets  *Taka* | If someone wanted to buy this enterprise today, how much would they have to pay?  *Taka* |
| --- | --- | --- | --- | --- | --- | --- | --- | --- | --- | --- | --- | --- | --- | --- |
|  |  |  |  | Wages | Rent | Raw Materials | Fuel, kerosene, electricity | Finished goods purchased for reselling | Other operating expenses |  |  |  |  |  |
|  | **G2_13** | **G2_14** | **G2_15** | **G2_16a** | **G2_16b** | **G2_16c** | **G2_16d** | **G2_16e** | **G2_16f** | **G2_17a** | **G2_17b** | **G2_18** | **G2_19** | **G2_20** |
| **1** |  |  |  |  |  |  |  |  |  |  |  |  |  |  |
| **2** |  |  |  |  |  |  |  |  |  |  |  |  |  |  |

**Module G2 Codes:**

| **noyes:** | |
| --- | --- |
| 0 | No |
| 1 | Yes |

| **kind_ent:** | |
| --- | --- |
| 1 | Fresh food seller |
| 2 | Convenience store (food items) |
| 3 | Restaurant/Tea Stall |
| 4 | Other food (e.g. processing, livestock) |
| 5 | Transportation service |
| 6 | Selling non-food products (e.g. household goods, wood, plastic, metal, medicines, cigarettes etc.) |
| 7 | Phone services |
| 8 | Financial services |
| 9 | Other services (e.g. construction, barber, mechanics, etc.) |
| 10 | Other (specify) |

| **place_ent:** | |
| --- | --- |
| 1 | Own house |
| 2 | Rented house |
| 3 | Govt land/house |
| 4 | Fixed location outside house |
| 5 | Variable location |

| **customer:** | |
| --- | --- |
| 1 | Households/Individuals |
| 2 | Government or other public firm |
| 3 | Private enterprises |
| 4 | Foreign individual /organization |
| 5 | Others |

| **source_finance:** | |
| --- | --- |
| 1 | Inherited/ through gift |
| 2 | Own savings |
| 3 | Borrowing from relatives/friends |
| 4 | Agricultural development bank |
| 5 | Commercial bank |
| 6 | Grameen bank |
| 7 | Other financial institution |
| 8 | NGO/ Relief agency |
| 9 | Sale of assets |
| 10 | Supplier's credit |
| 11 | Other |

| **problems_busi:** | |
| --- | --- |
| 1 | No problem |
| 2 | Inadequate capital or credit |
| 3 | Inadequate technology knowledge |
| 4 | Lack of required no. of expertise |
| 5 | High-cost of running the enterprise |
| 6 | Water supply problem |
| 7 | Power supply problem |
| 8 | Problems with equipment/ spare parts |
| 9 | Government regulations |
| 10 | Lack of raw materials |
| 11 | Lack of customers |
| 12 | Transport problems |
| 13 | Telecom and internet access |
| 14 | Other (specify) |

## **Module G3: Economic Activities and Wage Employment**

|  | **Question** | **Note** | **Response** |
| --- | --- | --- | --- |
| **G3_1_pre** | **Did you or anyone in your household undertake any wage employment the last 12 months?** | **Code [noyes]**  *(if no (0), skip to Module G4)* |  |
| **G3_1** | **Who had any wage employment in the past 12 months?** | *(Select all household members who apply and go through loop for each selected)* |  |
| **G3_1b** | **Did (member of HH) work in the last 30 days?** | **Code [noyes]** |  |

| Job ID | HH Mem ID | What economic activities did (name) do in the past 12 months?  **Code [occupation]**  *Select all that apply* | Which two are the most important?  **Code [occupation]** (filtered for those selected in G3_1c)  *(Continue loop only for these 2)* | How many months did (person) do this activity in the last 12 months? | On average, how many days per month? | On average how many hours per day? | Where did you do this activity?  **Code [urbanrural]** | Where exactly?  **Code [district_word]** | What was your employment status?  **Code [emp_status]**  *If self-employed or employer, skip to Module G4* |
| --- | --- | --- | --- | --- | --- | --- | --- | --- | --- |
|  |  | **G3_2** | **G3_2b** | **G3_3** | **G3_4** | **G3_5** | **G3_6a** | **G3_6b** | **G3_8** |
| 1 |  |  |  |  |  |  |  |  |  |
| 2 |  |  |  |  |  |  |  |  |  |

| Job ID | HH Mem ID | Were you paid on a daily basis?  **Code [noyes]**  *(if no (0), skip to G3_14)* | What was the daily wage in cash in the past 12 months?  *Taka* | | | Did you receive payments in-kind?  **Code [noyes]**  *(if no (0), skip go G3_14)* | What kind of payment did you receive?  **Code [paymentinkind]** | How much did you receive per day? | | What type of org. did/do you work for?  **Code [type_org_work]** | What is your gross remuneration per month?  *Taka* | What is your total net take-home monthly remuneration after all deductions and additions at source, including in-kind payment and other benefits (tips, bonuses)?  *Taka* |
| --- | --- | --- | --- | --- | --- | --- | --- | --- | --- | --- | --- | --- |
|  |  |  | Highest | Lowest | Average |  |  | Quantity in kg | Value in taka |  |  |  |
|  |  | **G3_9** | **G3_10a** | **G3_10b** | **G3_10c** | **G3_11** | **G3_12** | **G3_13a** | **G3_13b** | **G3_14** | **G3_15** | **G3_16** |
| 1 |  |  |  |  |  |  |  |  |  |  |  |  |
| 2 |  |  |  |  |  |  |  |  |  |  |  |  |

**Module G3 Codes:**

| **noyes:** | |
| --- | --- |
| 0 | No |
| 1 | Yes |

| **occupation:** | | | |
| --- | --- | --- | --- |
| 1 | Student | 39 | Plumber |
| 2 | Housewife | 40 | Electrician |
| 3 | Jobless | 41 | Carpenter |
| 4 | Retired | 42 | Mason |
| 5 | Child(age <5 no study/ work) | 43 | Doctor |
| 6 | Agricultural day labor | 44 | Village doctor |
| 7 | Earth work (government program) | 45 | Midwife |
| 8 | Earth work (other) | 46 | Herbal doctor/Kabiraj |
| 9 | Sweeper | 47 | Engineer |
| 10 | Scavenger | 48 | Lawyer/deed writer/Moktar |
| 11 | Tea garden worker | 49 | Religious leader (Imam/Muazzem/ Khadem/Purohit) |
| 12 | Construction labor | 50 | Lodging master |
| 13 | Factory worker | 51 | Private tutor/house tutor |
| 14 | Transport worker (bus/truck helper) | 52 | Beggar |
| 15 | Apprentice | 53 | Food Processing |
| 16 | Other wage labor (specify) | 54 | Small industry |
| 17 | Government/ parastatal | 55 | Handicrafts |
| 18 | Private enterprise (sales person, manager) | 56 | Small trader (roadside stand or stall) |
| 19 | NGO worker | 57 | Medium trader (shop or small store) |
| 20 | House maid | 58 | Large trader (large shop or whole sale) |
| 21 | Teacher (GoB-Primary school) | 59 | Fish Trader |
| 22 | Teacher(Non GoB Primary school) | 60 | Contractor |
| 23 | Teacher (GoB High school) | 61 | Milk collector |
| 24 | Teacher (Non-GoB High school) | 62 | Vet medicine seller |
| 25 | Teacher (college,university) | 63 | Feed supplier |
| 26 | Other salaried worker(specify) | 64 | Commercially feed producer |
| 27 | Rickshaw/van pulling | 65 | Animal Breeder |
| 28 | Driver of motor vehicle | 66 | Veterinary/paravet doctor |
| 29 | Tailor/seamstress | 67 | Working own farm (crop) |
| 30 | Potter | 68 | Share cropper/tenant |
| 31 | Blacksmith | 69 | Homestead farming |
| 32 | Cobbler | 70 | Fisherman (non owned/not leased water body) |
| 33 | Hair cutter | 71 | Raising fish / fish pond |
| 34 | Clothes washer | 72 | Raising poultry |
| 35 | Porter | 73 | Raising livestock |
| 36 | Goldsmith/silversmith | 74 | Milk producer |
| 37 | Repairman (appliances) | 75 | Other self employment |
| 38 | Mechanic (vehicles) | 76 | Physically/mentally challenged |

| **urbanrural:** | |
| --- | --- |
| 1 | Urban |
| 2 | Rural |

| **district_work:** | |
| --- | --- |
| 1 | Cox's Bazar |
| 2 | Chittagong |
| 3 | Bandarban |
| 4 | Rangamati |
| 5 | Other (specify) |

| **emp_status:** | |
| --- | --- |
| 1 | Day laborer |
| 2 | Self employed |
| 3 | Employer |
| 4 | Employee |

| **paymentinkind:** | |
| --- | --- |
| 1 | Paddy |
| 2 | Rice |
| 3 | Wheat |
| 4 | Meal |
| 5 | Other (specify) |

| **type_org_work:** | |
| --- | --- |
| 1 | Government organization |
| 2 | Autonomous body |
| 3 | Private office/institution |
| 4 | Public mill/ factory |
| 5 | Private mill/ factory |
| 6 | Local government |
| 7 | NGO |
| 8 | Household |
| 9 | Other (specify) |

## **Module G4: Other Income**

| **In the last 30 days, what have been the three main sources of income that have sustained your household** | |  |  |
| --- | --- | --- | --- |
| **G5_6a** | Main source | **Code [source_income]** |  |
| **G5_6b** | Second source *(If more than 1 sources)* | **Code [source_income]** |  |
| **G5_6c** | Third source *(If more than 2 sources)* | **Code [source_income]** |  |

|  | **Question** | **Note** | **Response** |
| --- | --- | --- | --- |
| **G4_1** | Did you receive any remittances from relatives in the last 12 months?  *(if no (0), skip to G4_4)* | **Code [noyes]** |  |
| **G4_2** | Remittances received from relatives during the past 12months: | Taka |  |
| **G4_3** | Where did you mainly invest/spend the received money? | **Code [where_invest]** |  |
| **G4_4** | Did you receive charity, gift, royalty, help, zakat, fitra or other such private (ie non-WFP) assistance in the last 12 months?  *(if no (0), skip to G4_7)* | **Code [noyes]** |  |
| **G4_5** | Charity, gift, royalty, help, zakat, fitra or other such assistance, etc. received during the past 12 months: IN CASH | Taka |  |
| **G4_6** | Charity, gift, royalty, help, zakat, fitra or other such assistance, etc. received during the past 12 months: IN-KIND | Value in Taka |  |
| **G4_7** | Did you receive any income from land rental, property, interest or dividends in the last 12 months?  *(if no (0), skip to G4_12)* | **Code [noyes]** |  |
| **G4_8** | Income from rent of land received during the past 12 months: | Taka |  |
| **G4_9** | Income from rent of other property received during the past 12 months: | Taka |  |
| **G4_10** | Profit and dividend received as partner/share holder during the past 12 months: | Taka |  |
| **G4_11** | Interest received during the past 12 months:  (FROM BANKS AND OTHER SOURCES) | Taka |  |
| **G4_12** | Did you receive any income from any other source not already mentioned? Examples include: social and life insurance, lotteries or other prizes, gratuities or retirement benefits  *(if no, skip to G4_16)* | **Code [noyes]** |  |
| **G4_13** | Social and insurance (life and non-life) income received during the past 12 months: | Taka |  |
| **G4_14** | Lottery/prize bond/ other similar income received in cash or in-kind during the past 12 months: | Taka |  |
| **G4_15** | Gratuity, separation payment, retirement benefit received during the past 12 months: | Taka |  |
| **G4_16** | Other cash or in-kind receipts during the past 12 months: (DO NOT INCLUDE RECEIPTS ALREADY REPORTED) | Taka |  |

**Module G4 Codes:**

| **where_invest:** | |
| --- | --- |
| 1 | Construction |
| 2 | Business |
| 3 | Education |
| 4 | Marriage |
| 5 | Consumption |
| 6 | Treatment |
| 7 | Other (Specify) |

| **noyes:** | |
| --- | --- |
| 0 | No |
| 1 | Yes |

# Module H: Credit

|  | **Question** | **Note** | **Response** |
| --- | --- | --- | --- |
| **H1_1** | During the past **3 months**, did any member of your household borrow money?  *(if no (0), skip to Module I)* | **Code [noyes]** |  |
| **H1_2** | What was the **primary** reason for borrowing money in the last three months? | **Code [reason_credit]** |  |
| **H1_2b** | What was the main **source** of that credit? | **Code [source_credit]** |  |
| **H1_3** | How much did you borrow in the last **3 months**? | Taka |  |
| **H1_4** | Is any part of these loans still outstanding?  *(if no (0), skip to Module I)* | **Code [noyes]** |  |
| **H1_5** | How many months will it take before you will have paid off all of these loans? | In months  *9997=don't know (person who knows is absent)*  *9998=not applicable (don't have a repayment period)* |  |

**Module H Codes:**

| **reason_credit:** | |
| --- | --- |
| 1 | Food |
| 2 | Shelter/rent |
| 3 | Firewood |
| 4 | Health |
| 5 | Other essential household needs |
| 6 | Social Event |
| 7 | Other (specify) |

| **source_credit:** | |
| --- | --- |
| 1 | Money Lender |
| 2 | Grocery Store inside the Camp |
| 3 | Grocery Store Outside the Camp |
| 4 | Employer inside the camp |
| 5 | Employer Outside the Camp |
| 6 | Co-operative Bank |
| 7 | Co-operative Association |
| 8 | Microfinance Institutions/NGO (Grameen Bank, BRAC, ASA etc.) |
| 9 | International Organizations |
| 10 | Commercial Bank outside the camp |
| 11 | Government of Bangladesh (for example, Bangladesh Rural Development Board) |
| 12 | Friends/Relatives Inside the Camp |
| 13 | Friends/Relatives in Myanmar |
| 14 | Friends/Relatives in Bangladesh but outside the camp |
| 15 | Friends/Relatives in Countries Other Than Bangladesh and Myanmar |
| 16 | Input Suppliers |
| 17 | Land Owner of my House |
| 18 | Other (specify) |

| **noyes:** | |
| --- | --- |
| 0 | No |
| 1 | Yes |

# Module I: Consumption Expenditure

## **Module I1: Food consumption**

| Name of the item | Code | Did your household consume it in the last 7 days?  **Code [noyes]**  *If no, skip to next item/line* | Total quantity consumed  *(If the item was consumed)* | Unit of measure?  **Code [measures]** | If the unit of measure is number, then write the average weight in grams | What was the total value of this food consumed in the last 7 days?  *Taka* | What was the value of this food consumed in the last 7 days…. | | | | Total quantity purchased on either cash or credit | Unit of measure?  **Code [measures]** | How much did you spend on purchases of [ITEM] outside the camp?  *Taka*  **(Rohingya only)** |
| --- | --- | --- | --- | --- | --- | --- | --- | --- | --- | --- | --- | --- | --- |
|  |  |  |  |  |  |  | Purchased in cash  *Taka* | Purchased on credit  *Taka* | Purchased using e-voucher  *Taka*  **(Rohingya only)** | Received in-kind  *(taka estimated value)* |  |  |  |
|  |  | I1_1 | I1_2 | I1_3 | I1_4 | I1_5 | I1_5a | I1_5b | I1_5c | I1_5d | I1_7 | I1_8 | I1_10 |
| Rice/Chira/Muri/Khoi/Rice flour/Suji (cream of wheat/barley) |  |  |  |  |  |  |  |  |  |  |  |  |  |
| Wheat, Atta and Maida (wheat flour w/o bran) |  |  |  |  |  |  |  |  |  |  |  |  |  |
| Semai/noodles |  |  |  |  |  |  |  |  |  |  |  |  |  |
| Other cereals |  |  |  |  |  |  |  |  |  |  |  |  |  |
| Lentil |  |  |  |  |  |  |  |  |  |  |  |  |  |
| Chickpea |  |  |  |  |  |  |  |  |  |  |  |  |  |
| Anchor daal |  |  |  |  |  |  |  |  |  |  |  |  |  |
| Mung |  |  |  |  |  |  |  |  |  |  |  |  |  |
| Other pulses | 1. 13 |  |  |  |  |  |  |  |  |  |  |  |  |
| Soybean |  |  |  |  |  |  |  |  |  |  |  |  |  |
| Mustard |  |  |  |  |  |  |  |  |  |  |  |  |  |
| Palm oil |  |  |  |  |  |  |  |  |  |  |  |  |  |
| Sesame oil |  |  |  |  |  |  |  |  |  |  |  |  |  |
| Other edible oil |  |  |  |  |  |  |  |  |  |  |  |  |  |
| Gourd |  |  |  |  |  |  |  |  |  |  |  |  |  |
| Okra |  |  |  |  |  |  |  |  |  |  |  |  |  |
| Eggplant |  |  |  |  |  |  |  |  |  |  |  |  |  |
| Tomato |  |  |  |  |  |  |  |  |  |  |  |  |  |
| Long bean and Sheem |  |  |  |  |  |  |  |  |  |  |  |  |  |
| Carrot |  |  |  |  |  |  |  |  |  |  |  |  |  |
| Cauliflower | 1. 37 |  |  |  |  |  |  |  |  |  |  |  |  |
| Green chili |  |  |  |  |  |  |  |  |  |  |  |  |  |
| Cucumber |  |  |  |  |  |  |  |  |  |  |  |  |  |
| Kachu (arum) |  |  |  |  |  |  |  |  |  |  |  |  |  |
| Potato |  |  |  |  |  |  |  |  |  |  |  |  |  |
| Onion |  |  |  |  |  |  |  |  |  |  |  |  |  |
| Garlic |  |  |  |  |  |  |  |  |  |  |  |  |  |
| Kachur lati |  |  |  |  |  |  |  |  |  |  |  |  |  |
| Jhinga (ribbed gourd) |  |  |  |  |  |  |  |  |  |  |  |  |  |
| Kolar mocha |  |  |  |  |  |  |  |  |  |  |  |  |  |
| Soybean bori |  |  |  |  |  |  |  |  |  |  |  |  |  |
| Kakrol |  |  |  |  |  |  |  |  |  |  |  |  |  |
| Pui (Indian spinach) |  |  |  |  |  |  |  |  |  |  |  |  |  |
| Shak (any) |  |  |  |  |  |  |  |  |  |  |  |  |  |
| Onion/garlic stalk |  |  |  |  |  |  |  |  |  |  |  |  |  |
| Leaves/leafy vegetables (any) |  |  |  |  |  |  |  |  |  |  |  |  |  |
| Other vegetables | 1. 83 |  |  |  |  |  |  |  |  |  |  |  |  |
| Beef/buffalo |  |  |  |  |  |  |  |  |  |  |  |  |  |
| Mutton |  |  |  |  |  |  |  |  |  |  |  |  |  |
| Chicken |  |  |  |  |  |  |  |  |  |  |  |  |  |
| Duck | 1. 115 |  |  |  |  |  |  |  |  |  |  |  |  |
| Dry Fish |  |  |  |  |  |  |  |  |  |  |  |  |  |
| Birds/bok/gughu/pigeon |  |  |  |  |  |  |  |  |  |  |  |  |  |
| Fish (large) |  |  |  |  |  |  |  |  |  |  |  |  |  |
| Fish (small) |  |  |  |  |  |  |  |  |  |  |  |  |  |
| Egg |  |  |  |  |  |  |  |  |  |  |  |  |  |
| Milk |  |  |  |  |  |  |  |  |  |  |  |  |  |
| Condensed Milk |  |  |  |  |  |  |  |  |  |  |  |  |  |
| Powdered Milk |  |  |  |  |  |  |  |  |  |  |  |  |  |
| Butter |  |  |  |  |  |  |  |  |  |  |  |  |  |
| Other meat |  |  |  |  |  |  |  |  |  |  |  |  |  |
| Mango |  |  |  |  |  |  |  |  |  |  |  |  |  |
| Banana |  |  |  |  |  |  |  |  |  |  |  |  |  |
| Papaya |  |  |  |  |  |  |  |  |  |  |  |  |  |
| Orange |  |  |  |  |  |  |  |  |  |  |  |  |  |
| Apple |  |  |  |  |  |  |  |  |  |  |  |  |  |
| Coconut and Green Coconut |  |  |  |  |  |  |  |  |  |  |  |  |  |
| Grapes |  |  |  |  |  |  |  |  |  |  |  |  |  |
| Amra | 1. 136 |  |  |  |  |  |  |  |  |  |  |  |  |
| Karambola |  |  |  |  |  |  |  |  |  |  |  |  |  |
| Jujube/dried jujube |  |  |  |  |  |  |  |  |  |  |  |  |  |
| Tamarind |  |  |  |  |  |  |  |  |  |  |  |  |  |
| Lemon |  |  |  |  |  |  |  |  |  |  |  |  |  |
| Sugarcane |  |  |  |  |  |  |  |  |  |  |  |  |  |
| Ata (bullock’s heart) |  |  |  |  |  |  |  |  |  |  |  |  |  |
| Tarmuj (Water melon) |  |  |  |  |  |  |  |  |  |  |  |  |  |
| Bangi (Musk melon) |  |  |  |  |  |  |  |  |  |  |  |  |  |
| Pineapple |  |  |  |  |  |  |  |  |  |  |  |  |  |
| Jaamrul |  |  |  |  |  |  |  |  |  |  |  |  |  |
| Other fruit (specify) |  |  |  |  |  |  |  |  |  |  |  |  |  |
| Dried chili |  |  |  |  |  |  |  |  |  |  |  |  |  |
| Turmeric |  |  |  |  |  |  |  |  |  |  |  |  |  |
| Salt |  |  |  |  |  |  |  |  |  |  |  |  |  |
| Other spices |  |  |  |  |  |  |  |  |  |  |  |  |  |
| Sugar |  |  |  |  |  |  |  |  |  |  |  |  |  |
| Gur |  |  |  |  |  |  |  |  |  |  |  |  |  |
| Tea leaves |  |  |  |  |  |  |  |  |  |  |  |  |  |
| Badam (ground nut) |  |  |  |  |  |  |  |  |  |  |  |  |  |
| Tea –prepared |  |  |  |  |  |  |  |  |  |  |  |  |  |
| Carbonated soda |  |  |  |  |  |  |  |  |  |  |  |  |  |
| Packaged Juice |  |  |  |  |  |  |  |  |  |  |  |  |  |
| Sugarcane/palm/date juice |  |  |  |  |  |  |  |  |  |  |  |  |  |
| Other beverages |  |  |  |  |  |  |  |  |  |  |  |  |  |
| Biscuit |  |  |  |  |  |  |  |  |  |  |  |  |  |
| Tobacco |  |  |  |  |  |  |  |  |  |  |  |  |  |
| Betel leaf |  |  |  |  |  |  |  |  |  |  |  |  |  |
| Betel Nut |  |  |  |  |  |  |  |  |  |  |  |  |  |

**Module I1 Codes:**

| **measures:** | |
| --- | --- |
| 1 | Kg |
| 2 | Grams |
| 3 | Liter |
| 4 | Number |

| **noyes:** | |
| --- | --- |
| 0 | No |
| 1 | Yes |

## **Module I2: Non-food Expenditure Annual Recall (October 1, 2017 – September 30, 2018)**

### Sub-module I2a: Non-food Expenditure Monthly Recall

|  |  | Did your household purchase or receive this item in the last month?  **Code [noyes]**  *(if no (0), skip to next item/line; except for items 1 and 2, which skip to I2a_5)* | If purchased/received | | | | | If **not** purchased/received | |
| --- | --- | --- | --- | --- | --- | --- | --- | --- | --- |
|  |  |  | What was the value of [item] purchased or received by your household in the last month… | | | | How much did you spend on purchases of [ITEM] outside the camp?  *Taka*  **(Rohingya only)** |  |  |
|  |  |  | Purchased in cash  *Taka* | Purchased on credit  *Taka* | Purchased using e-voucher  *Taka*  **(Rohingya only)** | Received in-kind  *(taka estimated value)* |  | Did you collect or produce this item yourself?  **Code [noyes]**  (if no (0)*, skip to the next item/line)* | What is the value of the item that you produced or collected yourself  *Taka* |
| **Item Name** | **code** | **I2a __1** | **I2a __2a** | **I2a __2b** | **I2a_2c** | **I2a __2d** | **I2a_3** | **I2a_5** | **I2a_6** |
| **FUEL AND LIGHTING** |  |  |  |  |  |  |  |  |  |
| Firewood |  |  |  |  |  |  |  |  |  |
| Cow dung/cakes/bhushi/wood-powder |  |  |  |  |  |  |  |  |  |
| Kerosene |  |  |  |  |  |  |  |  |  |
| Electricity |  |  |  |  |  |  |  |  |  |
| Other fuels and light (e.g. matches and candles etc.) |  |  |  |  |  |  |  |  |  |
| **TOILETRIES AND CLEANING EXPENSES** |  |  |  |  |  |  |  |  |  |
| Cosmetics |  |  |  |  |  |  |  |  |  |
| Bath soap, shampoo, toothpaste, etc. |  |  |  |  |  |  |  |  |  |
| Washing soap, powder for cloths |  |  |  |  |  |  |  |  |  |
| Mosquito coil |  |  |  |  |  |  |  |  |  |
| **TRANSPORT/ TRAVEL AND OTHER MISC. CHARGES** |  |  |  |  |  |  |  |  |  |
| Bus fare |  |  |  |  |  |  |  |  |  |
| Rickshaw/ van fare |  |  |  |  |  |  |  |  |  |
| Taxi/ tempo/ mishuk fare |  |  |  |  |  |  |  |  |  |
| Boat/ launch fare |  |  |  |  |  |  |  |  |  |
| Other transport fare (specify) |  |  |  |  |  |  |  |  |  |
| Bicycle maintenance, tires, tubes repairs etc. |  |  |  |  |  |  |  |  |  |
| Other transport, repair and maintenance. |  |  |  |  |  |  |  |  |  |
| Telephone bill/ charges/mobile |  |  |  |  |  |  |  |  |  |

### Sub-module I2b: Non-food Expenditure Annual Recall (October 1, 2017 – September 30, 2018)

|  |  | Did you purchase/receive this item in the last year?  **Code [noyes]**  *(if no (0), skip to next item/line)* | How many did you buy/receive? | What was the value of [item] purchased or received by your household in the last year… | | | | How much did you spend on purchases of [ITEM] outside the camp?  *Taka*  **(Rohingya only)** |
| --- | --- | --- | --- | --- | --- | --- | --- | --- |
|  |  |  |  | Purchased in cash  *Taka* | Purchased on credit  *Taka* | Purchased using e-voucher  *Taka*  **(Rohingya only)** | Received in-kind  *(taka estimated value)* |  |
| **Item Name** | **Code** | **I2b _1** | **I2b _2** | **I2b _3a** | **I2b_3b** | **I2b_3c** | **I2b_3d** | **I2b_4** |
| **CLOTHES** |  |  |  |  |  |  |  |  |
| Lungi/dhuti |  |  |  |  |  |  |  |  |
| Shirts |  |  |  |  |  |  |  |  |
| Pants |  |  |  |  |  |  |  |  |
| Sarees |  |  |  |  |  |  |  |  |
| Blouse/ petticoat |  |  |  |  |  |  |  |  |
| Salwar kameez/ Orna |  |  |  |  |  |  |  |  |
| Punjabi/ Pajamas |  |  |  |  |  |  |  |  |
| Sweaters, Jacket, pullovers, mufflers, etc. |  |  |  |  |  |  |  |  |
| Underwear etc. |  |  |  |  |  |  |  |  |
| Socks, handkerchiefs, scarves, caps, neckties etc. |  |  |  |  |  |  |  |  |
| Frocks, dresses, baby suit etc. |  |  |  |  |  |  |  |  |
| **Bath** |  |  |  |  |  |  |  |  |
| Towel, Gamcha |  |  |  |  |  |  |  |  |
| Chador, shawl, etc. |  |  |  |  |  |  |  |  |
| **CLOTHING MATERIAL AND TAILORING** |  |  |  |  |  |  |  |  |
| Tailoring and related expenses |  |  |  |  |  |  |  |  |
| **FOOTWEAR** |  |  |  |  |  |  |  |  |
| Leather and plastic shoes/sandals |  |  |  |  |  |  |  |  |
| Maintenance and repair expenses of foot wear |  |  |  |  |  |  |  |  |
| **HOUSEHOLD-USE TEXTILES, ETC.** |  |  |  |  |  |  |  |  |
| Quilt/blanket/Katha |  |  |  |  |  |  |  |  |
| Bedsheets |  |  |  |  |  |  |  |  |
| Pillows, cushions |  |  |  |  |  |  |  |  |
| Pillow cover, cushion cover |  |  |  |  |  |  |  |  |
| Mosquito netting |  |  |  |  |  |  |  |  |
| Other (specify) |  |  |  |  |  |  |  |  |
| **HOUSING RELATED EXPENSES** |  |  |  |  |  |  |  |  |
| Home additions/ improvements |  |  |  |  |  |  |  |  |
| Routine maintenance/ repair |  |  |  |  |  |  |  |  |
| **MEDICAL TREATMENT EXPENSES** |  |  |  |  |  |  |  |  |
| Doctor's and other practitioner’s fees |  |  |  |  |  |  |  |  |
| Hospitalization |  |  |  |  |  |  |  |  |
| Medicines |  |  |  |  |  |  |  |  |
| Medical Tests (X-ray, blood, urine etc.) |  |  |  |  |  |  |  |  |
| Health-related travel/ incidental expenses |  |  |  |  |  |  |  |  |
| **EDUCATIONAL EXPENSES** |  |  |  |  |  |  |  |  |
| Registration and annual fees |  |  |  |  |  |  |  |  |
| Monthly fees |  |  |  |  |  |  |  |  |
| Examination fees |  |  |  |  |  |  |  |  |
| Personal Teaching expenses |  |  |  |  |  |  |  |  |
| Text book/ note books/ stationary |  |  |  |  |  |  |  |  |
| Other |  |  |  |  |  |  |  |  |
| **REMITTANCES, CEREMONIES, GIFTS, ETC.** |  |  |  |  |  |  |  |  |
| Fitra and donation/sadqa |  |  |  |  |  |  |  |  |
| Qurbani |  |  |  |  |  |  |  |  |
| Religious functions (milad etc.) |  |  |  |  |  |  |  |  |
| Expenditure on marriage |  |  |  |  |  |  |  |  |
| Other (specific) |  |  |  |  |  |  |  |  |
| **RECREATION & LEISURE, ETC.** |  |  |  |  |  |  |  |  |
| Photographs, books and magazines |  |  |  |  |  |  |  |  |
| Other leisure related expenses |  |  |  |  |  |  |  |  |
| **COOKING EQUIPMENT** |  |  |  |  |  |  |  |  |
| Glass/china/clay plates and dishes etc. |  |  |  |  |  |  |  |  |
| Pots/ pans |  |  |  |  |  |  |  |  |
| Other kitchen ware and utensils |  |  |  |  |  |  |  |  |
| Spoons/ forks/ knives etc. |  |  |  |  |  |  |  |  |
| FURNITURE |  |  |  |  |  |  |  |  |
| Khat/Choki |  |  |  |  |  |  |  |  |
| Table/Chair/Dressing Table |  |  |  |  |  |  |  |  |
| Trunk/Suitcase |  |  |  |  |  |  |  |  |
| Other furniture related expenses |  |  |  |  |  |  |  |  |
| **PERSONAL ARTICLES** |  |  |  |  |  |  |  |  |
| Gold and Silver Jewelry |  |  |  |  |  |  |  |  |
| Imitation Jewelry |  |  |  |  |  |  |  |  |
| Umbrella, walking stick |  |  |  |  |  |  |  |  |
| Other personal use items (belts, etc>) |  |  |  |  |  |  |  |  |
| **MISC. HOUSEHOLD DURABLE** |  |  |  |  |  |  |  |  |
| Lantern/ chimney lamp etc. |  |  |  |  |  |  |  |  |
| Electric fans, air-conditioners, coolers, etc. |  |  |  |  |  |  |  |  |

|  | **Question** | **Note** | **Response** |
| --- | --- | --- | --- |
| **I2b _5** | Please select if in the last year you received any of the following as gift  *(If none, skip to K1_1)* | **Code [non_food_giftyr]** |  |
| **I2b _6** | How many pieces (of object) did you receive as gift in the last year? |  |  |
| **I2b _7** | What is the value of the (object) that you received as gift in the last year? | Taka |  |

# Module K: Histories

## **Module K1: Migration history (Rohingya only)**

|  | **Question** | | **Note** | **Response** |
| --- | --- | --- | --- | --- |
| **K1_1** | What group does this household belong to? | | **Code [wave]** |  |
| **K1_2** | From where in Myanmar did your household arrive? | | **Code [origin_myan]** |  |
| **K1_2b** | Did you live in an urban or rural area in Myanmar? | | **Code [urbanrural]** |  |
| **The following questions are to be asked only to those who arrived after October 2016** | | | | |
| **K1_3** | How long did the travel take? | | *In days* |  |
| **K1_4** | How much did it cost for your household to travel (from starting point to final destination)? | Amount |  |  |
|  |  | Currency | **Code [currency]** |  |
| **K1_5** | How many household members were traveling together? | |  |  |
| **K1_6** | Did you bring anything when you came to Bangladesh?  *(if no (0), skip to K1_8)* | | **Code [noyes]** |  |
| **K1_7** | What were you able to bring? | | **Code [bring_myan]**  *Select all that apply* |  |
| **K1_8** | When you first crossed the border, which of the following were survival strategies for your family? | | Code [survival_strategy]  *Select all that apply* |  |
| **K1_9** | How long after arriving in Bangladesh did you settle in this block/sub-block? | | *Weeks* |  |
| **K1_10** | What percent of the people in your block/sub-block do you know from Myanmar? | | *Percentage* |  |
| **K1_11** | Did you choose to live in the block/sub-block? | | Code [noyes] |  |

**Module K1 Codes:**

| **wave:** | |
| --- | --- |
| 1 | Arrived after 25 August 2017 |
| 2 | Arrived between October 2016 and 24 Aug. 2017 |
| 3 | Arrived before October 2016 |

| **origin_myan:** | |
| --- | --- |
| 1 | Maungdaw |
| 2 | Buthidaung |
| 3 | Rathedaung |
| 4 | Other (specify) |

| **urbanrural:** | |
| --- | --- |
| 1 | Urban |
| 2 | Rural |

| **bring_myan:** | |
| --- | --- |
| 1 | Clothes |
| 2 | Kitchen items |
| 3 | Money/savings |
| 4 | Jewelry/gold |
| 5 | Electric devices (e.g. solar panel) |
| 6 | Chicken |
| 7 | Cow |
| 8 | Other (specify) |

| **survival_strategy:** | |
| --- | --- |
| 1 | Get relief |
| 2 | Seek employment |
| 3 | Remittances from relatives abroad |
| 4 | Help from relatives in Bangladesh |
| 5 | Sale assets/savings |
| 6 | Adopt even unwanted profession |

| **noyes:** | |
| --- | --- |
| 0 | No |
| 1 | Yes |

| **currency:** | |
| --- | --- |
| 1 | Bangladeshi Taka |
| 2 | Burmese Kyat |

## **Module K2: History prior to migration (Rohingya only)**

|  | Question | **Note** | **Response** |
| --- | --- | --- | --- |
| **K2_1** | How do you self-categorize your status in terms of food adequacy prior to your migration? | Code [foodadeq_myan] |  |
| **K2_2** | Could you travel freely outside of your village prior to your migration? | Code [travel_free] |  |
| **K2_2_livelihood** | Please describe the primary livelihood of this household before you were forced to migrate | Code [livelihood_myan] |  |
| **K2_3** | Cultivated land owned in the year prior to migration? | Decimals |  |
| **K2_4** | What were the primary crop grown?  *(If cultivated land is more than 0)* | Code [crops] |  |
| **K2_5** | Primary language spoken at home | Code [language] |  |
| **K2_7** | Does anyone in the household speak or understand Bangla (other dialect)? | Code [noyes] |  |
| **K2_8** | Does anyone in the household speak or understand Myanmar (Burmese)? |  |  |
| **K2_9** | Does anyone in the household speak or understand English? |  |  |

## **Module K3: Subjective wellbeing (ALL Households)**

| **“Thinking about your own household circumstances.”** | | | |
| --- | --- | --- | --- |
| **K3_1** | How would you describe your household in economic terms now? | **Code [subject_well]** |  |
| **K3_2** | How would you have described your household 1 year ago? |  |  |
| **K3_3** | How would you have described your household 5 years ago? |  |  |

**Module K2 and K3 Codes:**

| **foodadeq_myan** | |
| --- | --- |
| 1 | Always surplus |
| 2 | Sometimes surplus |
| 3 | Neither deficit nor surplus |
| 4 | Sometimes deficit |
| 5 | Always deficit |

| **crops:** | |
| --- | --- |
| 1 | Aus |
| 2 | Aman |
| 3 | Boro |
| 4 | Wheat |
| 5 | Maize |
| 6 | Jute |
| 7 | Sugarcane |
| 8 | Pulses |
| 9 | Oil Seed |
| 11 | Byproduct of paddy |
| 12 | Byproduct of wheat |
| 13 | Byproduct of jute |
| 14 | Byproduct of sugarcane |
| 15 | Potato |
| 16 | Onion |
| 17 | Garlic |
| 18 | Ginger |
| 19 | Turmeric |
| 21 | Tomato |
| 22 | Brinjal |
| 23 | Green Banana/Green Papaya |
| 24 | Cauliflower/Cabbage |
| 25 | Pumpkin |
| 26 | Radish |
| 27 | Green Chili |
| 28 | Bean |
| 29 | Patal |
| 31 | Lady's Finger |
| 32 | Puisak |
| 33 | Other byproduct (specify) |
| 41 | Mango |
| 42 | Jackfruit |
| 43 | Ripe Banana |
| 44 | Ripe Papaya |
| 45 | Pineapple |
| 46 | Leechee |
| 47 | Melon/Bangi |
| 48 | Guava |
| 49 | Other fruit (specify) |

| **travel_free** | |
| --- | --- |
| 0 | No |
| 1 | Yes (without permission) |
| 2 | Yes (only with permission) |

| **livelihood_myan:** | |
| --- | --- |
| 1 | Farming household |
| 2 | Landless labourer |
| 3 | Operated own business |
| 4 | Wage employment outside of agriculture |
| 5 | Other (specify) |

| **language:** | |
| --- | --- |
| 1 | Rohingya |
| 2 | Bangla |
| 3 | Burmese |
| 4 | English |
| 5 | Other (specify) |

| **subject_well** | |
| --- | --- |
| 1 | Very rich |
| 2 | Rich |
| 3 | Comfortable |
| 4 | Just enough to get by |
| 5 | Almost enough |
| 6 | Poor |
| 7 | Destitute |

| **noyes:** | |
| --- | --- |
| 0 | No |
| 1 | Yes |

# Module L: Protection (All households)

| **L1_1** | Have you heard of others in the current location being affected by any kind of insecurity other the last 3 months?  *(if no (0), skip to Module M)* | | | **Code [noyes]** |  |
| --- | --- | --- | --- | --- | --- |
| If yes, what kind? And who was mainly affected? **(Do not read the items one by one; all respondents to free list)** | | | | | |
|  | | **Did they list this one?**  **Code [noyes]** | **Who is mainly affected?**  **Code [mainly_affected]** | | |
|  | | **L1_2** | **L1_3** | | |
| 1. harassment | |  |  | | |
| 2. discrimination | |  |  | | |
| 3. theft/robbery | |  |  | | |
| 4. being approached by human smugglers | |  |  | | |
| 5. being approached by drug traffickers | |  |  | | |
| 6. physical violence/abuse | |  |  | | |
| 7. abduction | |  |  | | |
| 8. limitations on movement | |  |  | | |
| 9. house, land property destruction | |  |  | | |
| 10. Lost child (more than 1 day) | |  |  | | |
| 11. general unsafe feeling | |  |  | | |
| 12. tensions between the displaced and the host community | |  |  | | |
| 13. misuse of food and or nutrition assistance | |  |  | | |
| 14. if others, specify: | |  |  | | |

**Module L Codes:**

| **mainly_affected:** | |
| --- | --- |
| 1 | Adult females (18+) |
| 2 | Females (<18) |
| 3 | All females |
| 4 | Adult males (18+) |
| 5 | Males (<18) |
| 6 | All males |
| 7 | All |

| **noyes:** | |
| --- | --- |
| 0 | No |
| 1 | Yes |

# Module M: Assistance

## **Module M1: Assistance, Bangladesh (host) households**

|  |  | In the last 12 months, has anyone in your household received benefit from …?  Code [noyes]  *(if no (0), skip to next item/line)* | In the last month, how much did you receive in the form of: | | |
| --- | --- | --- | --- | --- | --- |
|  |  |  | Cash  *Taka* | Rice/Paddy  *Kg* | Wheat/Atta  *Kg* |
| **Code** | **Description** | **M1_1** | **M1_2** | **M1_3** | **M1_4** |
| **01** | Primary Education Stipend Program |  |  |  |  |
| **02** | Vulnerable Group Feeding (VGF) |  |  |  |  |
| **03** | Stipend for Secondary Education Female Students |  |  |  |  |
| **04** | Old Age Allowance |  |  |  |  |
| **05** | Gratuitous Relief (GR) |  |  |  |  |
| **06** | General Relief (GR) |  |  |  |  |
| **07** | Open market Sales (OMS) |  |  |  |  |
| **08** | Vulnerable Group Development (VGD) |  |  |  |  |

## **Module M2: Assistance, Rohingya households**

|  | **Description** | In the past month, did any member of your households benefit from...?  Code [noyes]  *(if no (0), skip to next item/line)* | Main assistance provider source  Code [provider_assistance]  *Select up to 2* |
| --- | --- | --- | --- |
|  |  |  |  |
| **code** |  | **M2_2** | **M2_3** |
| **01** | Cash transfers |  |  |
| **02** | Food for learning |  |  |
| **03** | High energy biscuits |  |  |
| **04** | Hot meals (khicuri) |  |  |
| **05** | Hygiene kit |  |  |
| **06** | Dignity kit or other clothes |  |  |
| **07** | Shelter |  |  |
| **08** | None |  |  |

|  | **Question** | **Note** | **Response** |
| --- | --- | --- | --- |
| **M2_5** | Did any member of your household benefited from any **nutrition intervention**?  *(if no (0), skip to M2_1a)* | **Code [noyes]** |  |
| **M2_6** | Which product did your household receive?**(Circle all that apply)** | **Code [product_nutrition]** |  |
| **M2_7** | Were any of your children screened? (MUAC)  *(if no (0), skip to M2_1a)* | **Code [noyes]** |  |
| **M2_8** | If so, what color category was it? (MUAC) | **Code [category_screened]** |  |

We would now like to ask you some questions about your experiences with the last payment that you received from the World Food Programme (e vouchers or food rations)

|  | **Question** | **Note** | **Response** |
| --- | --- | --- | --- |
| **M2_1a** | Do you receive in-kind food distribution (GFD) or cash/e-voucher?  *(if cash, skip to M2_1c)* | **Code [cashorfood]** |  |
| **M2_1b** | Which GFD option are you entitled to? | **Code [gfd_type]** |  |
| **M2_1c** | How frequently do you receive the food aid ration or e-voucher top of? | In days |  |
| **M2_1d** | How many days did the food from the general food aid ration or the e-voucher top-off from the last cycle last?  *If M2_1d is greater than or equal to M2_1c, skip to M2_9* | In days |  |
| **M2_1e** | What was the main reason why the general ration did not last until the next distribution? | **Code [food_last_cycle]** |  |
| **M2_9** | As best you can remember, what was the exact date that you were received your food ration (GFD) or top up on your e-voucher? |  |  |
| **M2_10** | How far did you have to travel to reach the site where this payment was made? (one way) | Meters and Km |  |
| **M2_11** | How did you return home from the payment site? | **Code [get_paymentsite]** |  |
| **M2_12** | How long did it take to travel home from the payment site? (one way) | Minutes and hours |  |
| **M2_13** | Were you subject to any harassment (verbal, physical, sexual) traveling to or returning from the payment site? | **Code [noyes]** |  |
| **M2_14** | Were you robbed of any food or cash while travelling to or returning from the payment site? | **Code [noyes]** |  |

We now want to ask you about how you used the last payment that you received. Just to be sure that we’ve understood this properly:

| ***CASH/E-VOUCHER Questions*** *(based on answer to M2_1a)* | | | |
| --- | --- | --- | --- |
|  | **Question** | **Note** | **Response** |
| **M2_16** | How much were you topped up? | Taka |  |
| **M2_17** | On the day you were paid, did anyone in a position of authority ask you to share this transfer with another household?  *(if no (0), skip toM2_20)* | **Code [noyes]** |  |
| **M2_18** | How many households did you share your transfers with? |  |  |
| **M2_19** | How much (Taka) did you give in total to all these other households (**not** to each household)? (If none, write “0”) | Taka |  |
| **M2_20** | On the day you were paid, did you give any money to anyone in a position of authority?  *(if no (0), skip to M2_22)* | **Code [noyes]** |  |
| **M2_21** | How much did you give to these persons in positions of authority? | Taka |  |
| **M2_22** | Who normally decides how to utilize cash in the family? | **Code [hh_decisions]** |  |
| **M2_23** | As of today, how much of this last transfer have you spent on food? | Taka |  |
| **M2_25** | Did you have any difficulties using your e-voucher to purchase food?  *(if no (0), skip to M2_35)* | **Code [noyes]** |  |
| **M2_26** | What were these difficulties?  *Allow for multiple responses* | **Code [difficulties_evou]** |  |

| ***IN-KIND/GFD Questions*** *(based on answer to XXX)* | | | | |
| --- | --- | --- | --- | --- |
|  | **Question** | | **Note** | **Response** |
| **M2_27** | How much of each food item did you receive in your most recent ration? | Grain/rice | kg |  |
|  |  | Pulse/lentil | kg |  |
|  |  | Oil | liter |  |
| ***The next questions will specifically be about the grain/rice that you received:*** | | | | |
| **M2_27d** | Was this amount of grain your whole quota for this distribution period? | | **Code [noyes]** |  |
| **M2_28** | On the day you were paid, did anyone in a position of authority ask you to share this transfer with another household?  *(if no (0), skip to M2_31)* | | **Code [noyes]** |  |
| **M2_29** | How many households did you share your transfers with? | |  |  |
| **M2_30** | How much (Kg) did you give in total to all these other households (**not** to each household)? (If none, write “0”) | | kg |  |
| **M2_31** | On the day you were paid, did you give any grain to anyone in a position of authority?  *(if no (0), skip to M2_33)* | | **Code [noyes]** |  |
| **M2_32** | How much (Kg) did you give to these persons in positions of authority? | | Kg |  |
| **M2_33** | Who normally decides how to utilize **food** in the family? | | **Code [hh_decisions]** |  |

| ***The next questions are asked to BOTH cash and in-kind recipients*** | | | |
| --- | --- | --- | --- |
|  | **Question** | **Note** | **Response** |
| **M2_35** | How much grain/rice from/bought with this last transfer do you still have stored? | kg |  |
| **M2_36a** | In the past month, did you sell or exchange food the grain/rice from the aid ration?  *Can select both sold (1) and gave/exchanged (2) if applicable* | **Code [sellaid]** |  |
| **M2_36b** | How much of this last transfer did you sell?  *If selected sold (1) in M2_36a* | kg of grain/rice |  |
| **M2_36c** | How much of this last transfer did you exchange?  *If selected gave/exchanged (2) in M2_36a* | kg of grain/rice |  |
| **M2_36d** | What did you exchange it for?  *If selected gave/exchanged (2) in M2_36a* | **Code [exchange_food]** |  |

Looking to the future

|  | **Question** | **Note** | **Response** |
| --- | --- | --- | --- |
| **M2_37a** | How often would you like to receive your payments/food? | **Code [period_payments]** |  |
| **M2_37b** | Why would you like to receive these payments/food at that frequency? | **Code [reason_period_payments]** |  |
| **M2_38** | What is your preferred modality of assistance to meet food, shelter and other basic needs?  ***(Definition of voucher:*** *token that will allow you to choose different food items from selected traders that would be close to your location)* | **Code [pref_cashevou]** |  |
| **M2_39** | Why this preference?  *Allow for multiple responses. (Enumerators don’t read)* | **Code [why_pref_cashevou]** |  |
| **M2_40** | How optimistic are you about your future? | **Code [aspir_resp]** |  |
| **M2_41** | How optimistic about your children’s future(s)? | **Code [aspir_kids]** |  |

| **pref_cashevou:** | |
| --- | --- |
| 1 | Food ration (GFD) |
| 2 | E-vouchers (Smart cards) |
| 3 | Cash |
| 4 | Combination of these (specify) |

**Module M2 Codes:**

| **provider_assistance:** | |
| --- | --- |
| 1 | Government / army |
| 2 | UN agency |
| 3 | NGOs |
| 4 | Religious body |
| 5 | Relatives |
| 6 | Local host community |
| 7 | Private sector/chamber of commerce |
| 8 | Other, specify |

| **difficulties_evou:** | |
| --- | --- |
| 1 | Could not buy the food I wanted |
| 2 | The card was not accepted |
| 3 | Other (specify) |

| **sellaid:** | |
| --- | --- |
| 0 | No |
| 1 | Sold food aid |
| 2 | Exchanged/bartered food aid |

| **noyes:** | |
| --- | --- |
| 0 | No |
| 1 | Yes |

| **get_paymentsite:** | |
| --- | --- |
| 1 | Foot |
| 2 | Rickshaw |
| 3 | Tom Tom |
| 4 | Bycicle |
| 5 | Other (specify) |

| **product_nutrition:** | |
| --- | --- |
| 1 | Suji/pusti and oil (WSB+ and oil) |
| 2 | Suji/pusti (WSB++) for women (prevention) |
| 3 | Suji/pusti (WSB++) for children (treatment) |
| 4 | Plumpy nut (RUTF) |
| 5 | Other (specify) |

| **exchange_food:** | |
| --- | --- |
| 1 | Meat |
| 2 | Sugar |
| 3 | Milk |
| 4 | Rice/pasta/potatoes |
| 5 | Vegetables |
| 6 | Wheat |
| 7 | Firewood |
| 8 | Shoes/clothing |
| 9 | Mobile phone airtime |
| 10 | School materials, fees |
| 11 | Food for livestock |
| 12 | Bus fare/transport |
| 13 | Water |
| 14 | Hygiene articles |
| 15 | Cooking utensils |
| 16 | Detergent/soap |
| 17 | Electricity |
| 18 | Production tools |
| 19 | Other (specify) |

| **why_pref_cashevou:** | |
| --- | --- |
| 1 | I prefer cash because I can use it as I wish |
| 2 | Cash can be saved and accumulated for later use (buying assets) |
| 3 | The food ration is tasty |
| 4 | The food ration supports my family need of food I would not be able to buy the same (required)  quantity of food if I would get cash due increases in food prices |
| 5 | I can decide the use of food |
| 6 | Food is required for food security and cash is needed for buying other foods |
| 7 | Food is required for food security and cash is needed for buying non-food items |
| 8 | Food will support my family need of food and cash save cash and accumulate for future use  (such as buying assets, starting IGA, etc) |
| 9 | Other (specify) |

| **category_screened:** | |
| --- | --- |
| 0 | Green |
| 1 | Yellow |
| 2 | Red |
| 9997 | Don't know |

| **cashorfood:** | |
| --- | --- |
| 1 | Cash (e voucher) |
| 2 | Food (GFD) |
| 3 | Both |

| **gfd_type:** | |
| --- | --- |
| 1 | 1-3 household members |
| 2 | 4-7 household members |
| 3 | 8/8+ household members |

| **aspir_resp:** | |
| --- | --- |
| 1 | Not at all optimistic |
| 2 | Slightly optimistic |
| 3 | Optimistic |
| 4 | Very optimistic |

| **aspir_kids:** | |
| --- | --- |
| 1 | Not at all optimistic |
| 2 | Slightly optimistic |
| 3 | Optimistic |
| 4 | Very optimistic |
| 9998 | Respondent does not have any kids |

| **food_last_cycle:** | |
| --- | --- |
| 1 | Food was sold or exchanged |
| 2 | New arrivals joined |
| 3 | Shared with relatives |
| 4 | Ration not big enough |
| 5 | Give to livestock |
| 6 | Lost due to theft |
| 7 | Lost due to bad storage |
| 8 | Other (specify) |

| **period_payments:** | |
| --- | --- |
| 1 | Weekly |
| 2 | Every two weeks |
| 3 | Monthly |

| **reason_period_payments:** | |
| --- | --- |
| 1 | I can plan my expenditures better |
| 2 | It would allow me to use credit |
| 3 | It is safer and more convenient |
| 4 | Other (specify) |

| **hh_decisions:** | |
| --- | --- |
| 1 | Male members |
| 2 | Female members |
| 3 | Jointy (both male and female members) |
