# Supplementary material for: Food transfers, electronic food vouchers and child nutritional status among Rohingya children living in Bangladesh
Source: PLoS One. 2020 Apr 29;15(4):e0230457. doi: 10.1371/journal.pone.0230457 (PMC7190090; doi:10.1371/journal.pone.0230457)
Supplement: Attachment — Submitted filename: Comments PLOSONE.docx [file pone.0230457.s003.docx]

This is an interesting topic of current value. The paper is well written although more clarity is needed in places. Overall, this study misses out on any attempt to explain why there was an association. Whilst the authors acknowledge this it is important to know how these interventions are working, especially if the information is to inform future use.

Specific points below

**Introduction**

Generally, the introduction very short. It would be beneficial if the authors were to refer to some of the more recently published studies of cash and vouchers in humanitarian settings e.g. REFANI studies, Grellety, Concern/Cornell.

Also it would be good for some reference to be made here to other cash/voucher programmes in Bangladesh, if any, with any evidence.

What is the nutritional status of children in this population in this area?

Ln 1 Please check reference #1 is correct as I cannot find the data it is referring too. Reference 1 is a website and not a specific reference.

Ln 57 Could the authors state here that the e-voucher is unconditional (assuming it is).

**Setting and methods**

**Setting and sample**

I find the description of the survey could be better. It would be good to understand the objective of the primary survey and whether it was designed also to measure the research question of this paper (intentional study). Or whether this is a sub-analysis on data that was collected anyway. Who was responsible for what? Who did the data collection? Were standardisation methods used in training sessions? What was the sample size and how was it calculated?

Ln 84 please define the type of survey e.g. cross-sectional

Lns 88-89 I do not see the relevance of the following sentence “The survey also included Bangladeshi households living in the host community. However, they do not receive the food assistance discussed in this paper and so are excluded from our analysis.”

**Access to food assistance**

The authors mention a sample size of 523 children; it would be good to know how this was split between the different interventions

Ln 118 Reference 8 only states the categorisation of household sizes

Ln 118 and 119 “(After our survey was complete, WFP introduced a fourth category for households with more than 11 members [8])” – is this relevant to this study?

Ln 125 Please check if this reference (#8) is correct here

Ln 132-133 “At the time of the survey, across all households, 62%, received GFD, 34% received e-vouchers and 4% received both”. Does this refer to the original sample or the sub-sample? It would be a good idea to make sure information concerning the the original sample and sub-sample are clearly demarcated

How did the authors deal with these 4% of households that received both e-voucher and food?

It would be very interesting to know how long each family had had access to each transfer, whether this was different or not. At the same time was there any information on how the transfers had been used? E.g. Was food sold and the money used for other types of foods or non-food goods? Were e-vouchers exchanged for other (non-food) goods?

Ln 145-146 “Thus, while access to e-vouchers was not randomized and thus our study is associational, there is no evidence to suggest that access to e-vouchers was linked to specific child or household characteristics.” Was this assumption checked?

Ln 145 “Thus, while access to e-vouchers was not randomized and ***thus*** our study is associational...” I would argue that this is not the only reason and that non-randomised studies under the right conditions may offer more than association.

**Outcome measures**

Ln 151 Please check reference 9 is valid as it mentions references for school-aged children and adolescents

**Statistical analysis**

As mentioned above it would be good to know how the authors handled those households receiving both food and e-voucher

**Results**

The results are lacking the number of children in either group. Sample sizes would be useful here (as well as in the tables)

It would be good to present the data without stating clarifying information e.g. ‘relatively few’ and ‘few’. Especially as 17% is termed a ‘significant fraction’ and 29% termed ‘relatively few’.

Ln 199 food ratio should be food ration

Lns 199-202 Can this be re-written so not to use ‘less or more likely’ but rather e.g. ‘there were more girls than boys in the households receiving e-vouchers….etc.” The last sentence reads a bit odd and could be added to the preceding sentence

Lns 216-217 Please make clearer by adding that these results are for the whole sample

Lns 237-238 The authors say that “The magnitudes of these associations are small and are not statistically significant”. However, there are some significant differences that could be mentioned

**Discussion**

Ln 262 The authors write “Rather, receipt of an e-voucher is associated with **an increase** in HAZ of 0.38SD.” I would caution about inferring a trend here.

Ln 266 I suggest not to use the word ‘impact’ here as it infers causality

Lns 264-268 These two sentences could be clearer as I am not entirely sure what the authors are saying here. “Receipt of the e-voucher is associated with a lower risk of stunting, though this is imprecisely measured once we include a full set of controls. It is possible that this imprecision arises because, as Figure 1 suggests, the impact of e-vouchers, is concentrated on children with very low HAZ scores, well below the -2 cut off used to denote stunting”.

Ln 271 Change ‘on’ to ‘of’
