# Supplementary material for: Food transfers, electronic food vouchers and child nutritional status among Rohingya children living in Bangladesh
Source: PLoS One. 2020 Apr 29;15(4):e0230457. doi: 10.1371/journal.pone.0230457 (PMC7190090; doi:10.1371/journal.pone.0230457)
Supplement: Attachment — Submitted filename: Reviewer comment_vochure.docx [file pone.0230457.s004.docx]

    Comments to the Author

    The present study aimed to examine to examine associations between receipt of an electronic food voucher (e-voucher) compared to food rations on the nutritional status of Rohingya children living in refugee camps in Bangladesh. This is an important topic and data this area is scarce, making this a valuable contribution to the literature. Despite this strength, I have some suggestions for improvement.

    Abstract;

- I would recommend to rewrite the abstract for better clarity. For example, the phrase “…but this association is imprecisely measured: & “There is suggestive evidence that this association is larger for girls than boys”
- It is also good to describe the methodology in more detail. I am not sure what the study design the authors used and specific outcomes they are looking for.
- I strongly advise the authors to report results in clearer statements.

    Introduction:

- It would also be helpful for the authors to build more of a case for the correlates that they chose to examine here. The introduction need a major revision and shall cover some of the relevant published literature on the issue and need to be explicit enough to show the reader the gap in literature rather than just reporting “*little is known about the comparative effects of food and voucher payments on children’s nutritional staus*”
- The introduction could better be structured and expanded.
- It would also be helpful for the authors to build a concrete question mainly focusing on specific aspects of child nutrition (macro or micro)?

    Methods:

- Some additional detail and structure regarding the methods would be helpful. For example, any theoretical assumptions used to calculate their sample size? What was the anticipated difference in wasting and stunting between the groups? It is also better to be specific in the use of phrases such as chronic undernutrition could be better replaces with stunting and acute malnutrition with wasting etc
- Why would the authors restrict to sub samples given that they are the one who collected the data? Why only some covariates are measured in the subsample? Which covariates? The authors need to describe in details about this issue otherwise the study is liable to selection bias.
- It is better to structure the methods in to sub-sections such as sample size and sampling methods, interventions, measurements, quality control, data management and analysis etc.
- Please indicate and describe the main covariates collected in the study
- More information about measures employed by the authors to keep the quality of data needed.
- The authors need to justify why they included stunting as an outcome measure than other outcome measures that would be relevant in humanitarian settings. This is very important in a way that the main purpose of the support in humanitarian setting is not related to improve child growth rather to overcome acute food shortage.
- Any conceptual framework used to organize the data collection instrument?

    Results and Discussions

- Table 1 need to be revised as it is confusing for reader in this format. Please use a table with columns reporting number and frequencies for each covariate reported and disaggregated by the type of intervention, additional column reporting p-value. (please refer STROBE- papers reporting results from either RCT or case control studies)
- I am not sure the relevance of reporting the graphs 1 and 2. I feel that this is a duplication of reporting of results.
- The authors need to clearly report the result obtained from the regression output . Some of the terms or phrases they use may mislead readers, e.g. Line 233 “ .. *likelihood that the child is stunted but this association is only marginally statistically significant*” this is a misleading statement. It need to be a clear message to the reader that there is no association. (please also revise the abstract section not to misinform readers).
- The authors need to critically discuss on why would the intervention improve the HAZ score and but failed to be reflected in reduction of stunting prevalence? Is there any justification on this?
- The conclusion “ *Our results suggest that transitioning from food rations to electronic food vouchers does not adversely affect child nutritional status and may in fact be beneficial*” seems over reporting given the limitation in the study methodology. The authors may tone down the conclusion;
